# The dark genome in cardiovascular medicine

**DOI:** 10.1093/eurheartj/ehag514

**Published:** 2026-07-09

**Authors:** Despoina Kesidou, Simon D Brown, Lars Maegdefessel, Igor Ulitsky, Andrew H Baker

**Affiliations:** Institute for Neuroscience and Cardiovascular Research, Queens Medical Research Institute, University of Edinburgh, 47 Little France Crescent, Edinburgh EH16 4TJ, UK; Institute for Neuroscience and Cardiovascular Research, Queens Medical Research Institute, University of Edinburgh, 47 Little France Crescent, Edinburgh EH16 4TJ, UK; Institute of Molecular Vascular Medicine, TUM University Hospital, Munich 80802, Germany; Cluster for Nucleic Acid Sciences and Technologies—NUCLEATE, Institute of Molecular Vascular Medicine, TUM University Hospital, Munich 80802, Germany; Department of Immunology and Regenerative Biology and Department of Molecular Neuroscience, Weizmann Institute of Science, Rehovot 7610001, Israel; Institute for Neuroscience and Cardiovascular Research, Queens Medical Research Institute, University of Edinburgh, 47 Little France Crescent, Edinburgh EH16 4TJ, UK; Cardiovascular Research Institute Maastricht (CARIM) and Department of Pathology, University of Maastricht, Maastricht 6229HX, The Netherlands; MRC/BHF Centre of Research Excellence in Advanced Cardiac Therapies (REACT), Institute for Neuroscience and Cardiovascular Research, University of Edinburgh, 47 Little France Crescent, Edinburgh EH16 4TJ, UK

**Keywords:** Dark genome, Non-coding RNAs (ncRNAs), Cardiovascular disease, Transcriptomics, RNA therapeutics, Translational cardiovascular medicine

## Abstract

Only ∼1%–2% of the human genome directly codes for proteins. The remainder consists of non-coding DNA, often referred to as the ‘dark genome’. This includes regulatory elements, transposable and repetitive sequences, structural genomic features, pseudogenes, intronic and intergenic regions, and non-coding RNA (ncRNA) genes. These components are increasingly recognized as major regulators of gene expression, cell identity, and disease susceptibility. Currently, dark genome elements, particularly ncRNAs are increasingly recognized as important regulators of cardiovascular health and disease. Advances in genome analysis technologies have greatly improved our understanding of these non-coding regions and revealed clearer connections between the dark genome and cardiovascular traits. This review highlights major parts of the dark genome involved in cardiovascular disease, with emphasis on those for which mechanistic understanding and translational relevance are beginning to emerge. As mechanistic insight into individual and collective components of the dark genome advances, it increasingly enables the development of new opportunities for targeted therapeutics for cardiovascular prevention and disease management.

## Graphical Abstract

**Graphical Abstract:**
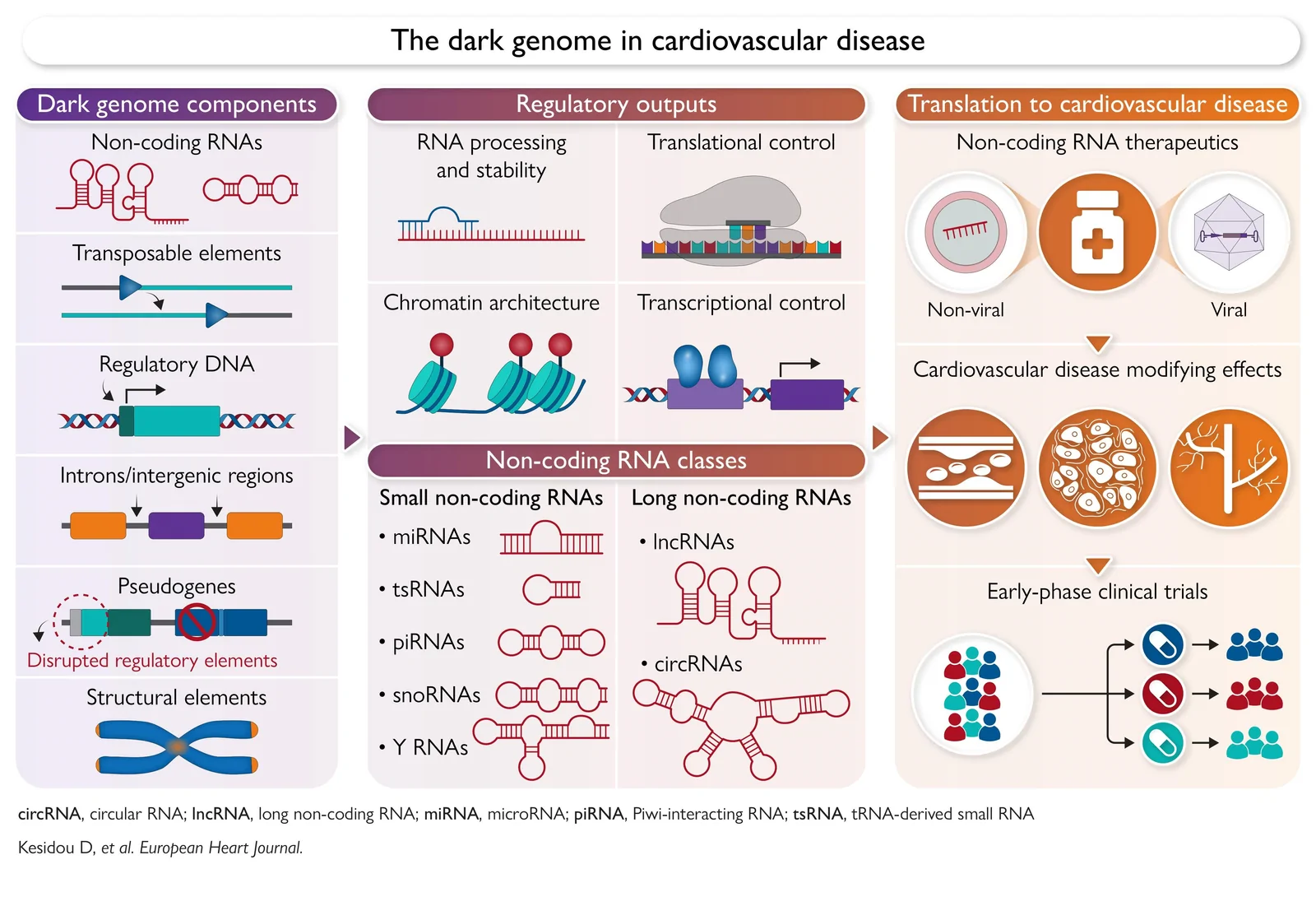
The dark genome landscape and therapeutic targeting in cardiovascular disease. Schematic overview of functional components within the dark genome. (Left) Non-coding RNAs, transposable elements, regulatory DNA regions (promoters, enhancers), introns and intergenic regions, pseudogenes and structural elements (telomeres, centromeres) modulate cardiovascular homeostasis. (Centre) Non-coding RNA species, including microRNAs, long non-coding RNAs (lncRNAs), circular RNAs (circRNAs), and other non-coding RNA species, serve as regulators modulating molecular processes at the transcriptional, post-transcriptional, translational and post-translational level. (Right) Pathological dysregulation of these dark genome elements drives cardiovascular disease progression, which can be therapeutically targeted to restore cardiovascular function. Ultimately, as a greater understanding of the dark genome emerges, novel target identification will enable a new class of advanced cardiovascular therapeutics to arrest or reverse disease progression as well as creating a wealth of new information.

## Introduction—What is the ‘dark genome’?

The genome contains the genetic blueprint required for an organism’s development and physiology. Protein-coding genes were traditionally considered the principal cellular mediators, and their importance in cardiovascular health is well-established.^1^ However, the genetic sequence corresponding to protein-coding genes accounts for only ∼1%–2% of the human genome.^2^ Previously, the remaining 98%–99% was considered non-functional or ‘junk’ DNA.^3^ Modern genomic studies have since revealed that biological complexity is instead encoded within non-coding regulatory elements and their transcriptional output.^4–6^

**Figure 1:**
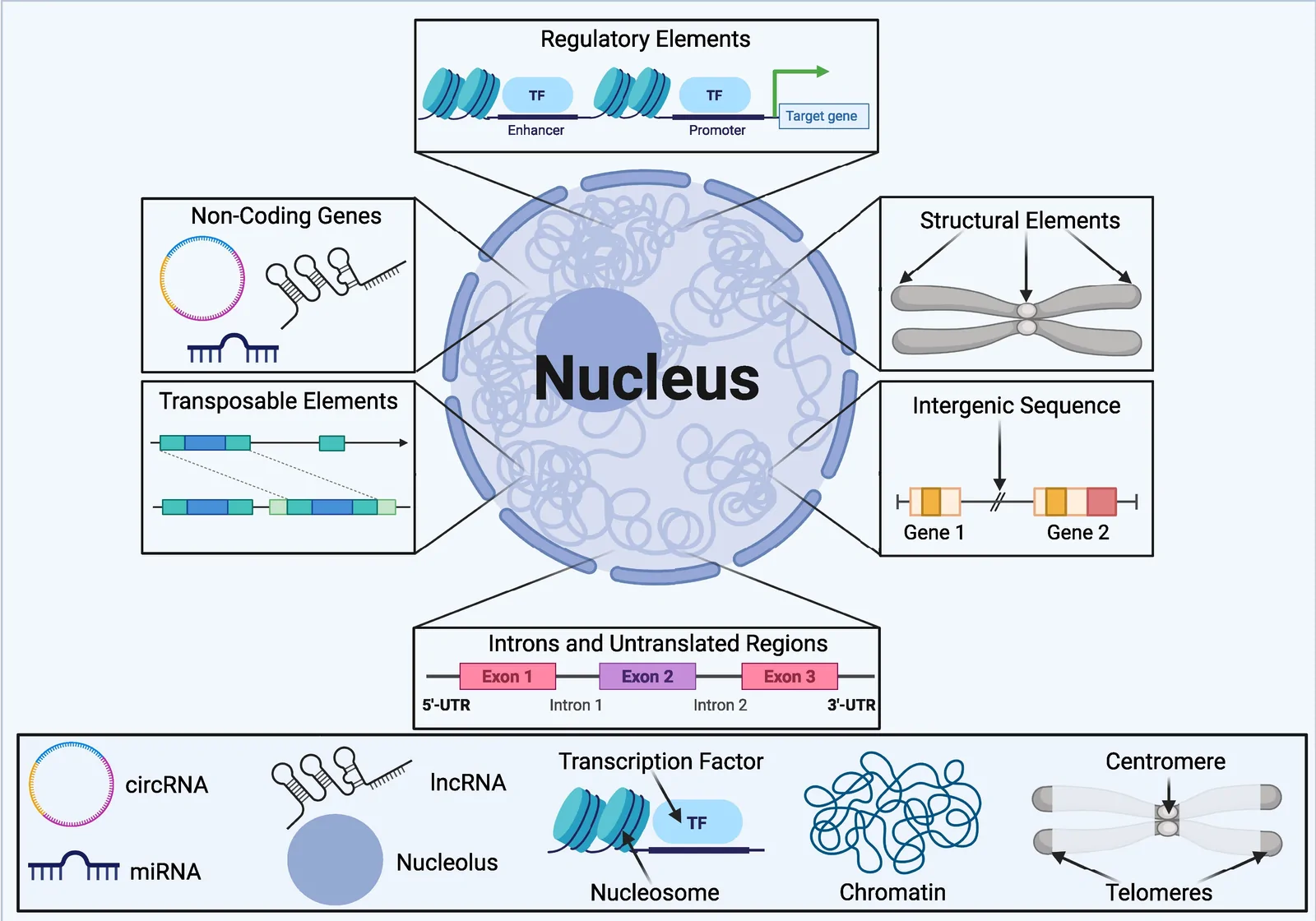
Make-up of the dark genome within the mammalian nucleus. Schematic overview of the major components of the dark genome and their organization within the mammalian nucleus. The dark genome comprises regulatory DNA elements (enhancers and promoters), structural elements (centromeres and telomeres), intergenic regions, introns and untranslated regions, transposable elements, and non-protein-coding genes that give rise to regulatory non-coding RNAs

The non-protein-coding DNA sequence of the genome consists of overlapping genomic elements collectively termed the ‘dark genome’ (*Figure 1*):

- Regulatory DNA elements (enhancers, silencers, and promoters)
- Structural elements (centromeres and telomeres)
- Introns
- Transposable elements
- Pseudogenes
- Intergenic regions
- Non-protein-coding genes

In this review, we discuss these components of the dark genome with emerging relevance to cardiovascular health and disease, summarizing experimental and computational approaches used for characterization, key methodological challenges, and future priorities for exploiting dark genome (patho)biology in cardiovascular research and medicine. A glossary of key terms, with definitions and relevance to the dark genome, is provided in Supplementary data online, *Table S1*.

## Dark genome regulatory layers in cardiovascular disease

In this section, we discuss major regulatory layers of the dark genome that are increasingly implicated in cardiovascular biology, including transposable elements, regulatory DNA elements, chromatin organization and structural features, pseudogenes, and RNA processing and splicing. While cardiovascular evidence varies, collective evidence illustrates the diverse ways in which these components can influence cardiovascular homeostasis and disease.

### Regulatory DNA elements

Enhancers and promoters are DNA elements that govern spatiotemporal control of gene expression. Promoters are DNA regions close to a gene’s start site that facilitate transcription initiation. Enhancers are regulatory DNA elements located distal to the transcription start site and increase gene transcription by bringing the enhancer and promoter into contact and recruiting transcription factors. In the healthy heart and vasculature, precise combinations of active enhancers and promoters define the gene expression programmes that establish and maintain cell identity.^7^ Variants in these regulatory regions are now recognized as central contributors to human cardiovascular disease.

**Figure 2:**
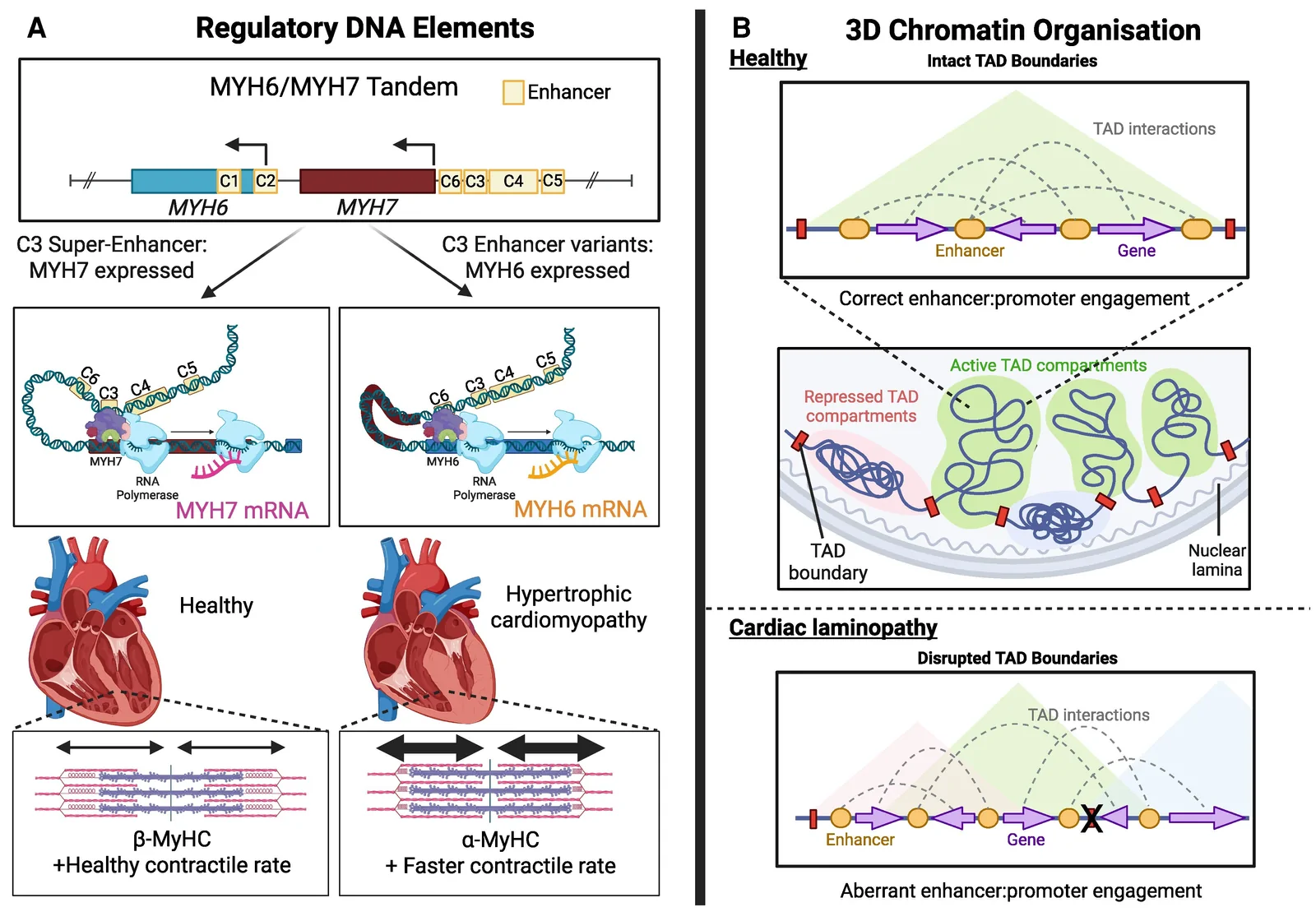
Regulatory elements of the dark genome: enhancers and higher-order chromatin structure. (*A*) Enhancer–promoter interactions. Transcription of the *MYH6*/*MYH7* tandem is regulated through the action of multiple enhancer sequences (C1–C6). Genetic variation in these enhancer sequences modifies *MYH6*/*MYH7* gene expression, leading to a dose-dependent increase in MYH6, a faster contractile rate in engineered cardiac tissues, and is associated with cardiomyopathy progression.^9^ (*B*) Higher-order, 3D chromatin organization is governed via topologically associated domains (TADs), which ensure correct gene expression patterns within chromosome territories. Mutations within TAD sequences can result in aberrant enhancer–promoter interactions and altered gene expression patterns, leading to diseases, such as cardiac laminopathies.^10^

Genome-wide profiling of human heart tissue revealed thousands of active promoters and enhancers with region-specific activity patterns, many of which differ between healthy and failing hearts.^8^ These observations highlight that changes in gene regulation are a feature of cardiac disease. In heart failure, ventricular tissue shows changes in gene regulation, including shifts in promoter use and enhancer activity, which are linked to altered expression of genes involved in contractile function and metabolism.^9^ Consistent with this regulatory mechanism contributing to inter-individual variation, common genetic variants in regulatory DNA regions have been linked directly to cardiomyopathy phenotypes. Gacita *et al*. (2021) reported that non-coding variants within enhancer elements controlling cardiomyopathy genes, including MYH7 and LMNA, modified cardiomyocyte gene expression and correlated with disease progression in human cohorts and engineered human cardiomyocytes (*Figure 2A*).^11^ This study illustrates that enhancer–promoter variation influences gene regulation in cardiovascular tissues and contributes to disease susceptibility, progression, and phenotypic heterogeneity.

#### Methods to identify regulatory elements and assess their functionality

Several complementary approaches are used to identify and quantify regulatory DNA element activity. Chromatin accessibility assays, such as assay for transposase-accessible chromatin sequencing, identify genomic regions that are open and transcriptionally active, highlighting likely enhancers and promoters.^12^ Chromatin immunoprecipitation sequencing can then help distinguish these elements based on characteristic histone marks associated with transcriptional activity.^13^ In parallel, cap analysis of gene expression sequencing maps transcription start sites and promoter usage at high resolution.^14^ These methods enable construction of regulatory maps; these techniques are now being applied to cardiovascular tissues to help define how enhancer and promoter activity is altered in disease.

### Chromatin organization and structural features

Beyond discrete regulatory elements, the dark genome also includes structural and organizational features that shape how the genome functions in space and time. The physical organization of the genome within the nucleus adds a further layer of regulatory complexity as enhancers and promoters communicate through three-dimensional chromatin looping, and this spatial organization is constrained by topologically associating domains (TADs): regions of the genome that preferentially interact with each other and are insulated from neighbouring domains.^15^ TAD boundaries help ensure that enhancers activate the correct target genes; when these boundaries are disrupted, enhancers can aberrantly activate genes in neighbouring domains, with potential pathological consequences.^15^ Alterations in three-dimensional chromatin organization have been described in cardiovascular diseases, where widespread changes in enhancer–promoter contacts accompany transcriptional reprogramming (*Figure 2B*).^10^

**Figure 3:**
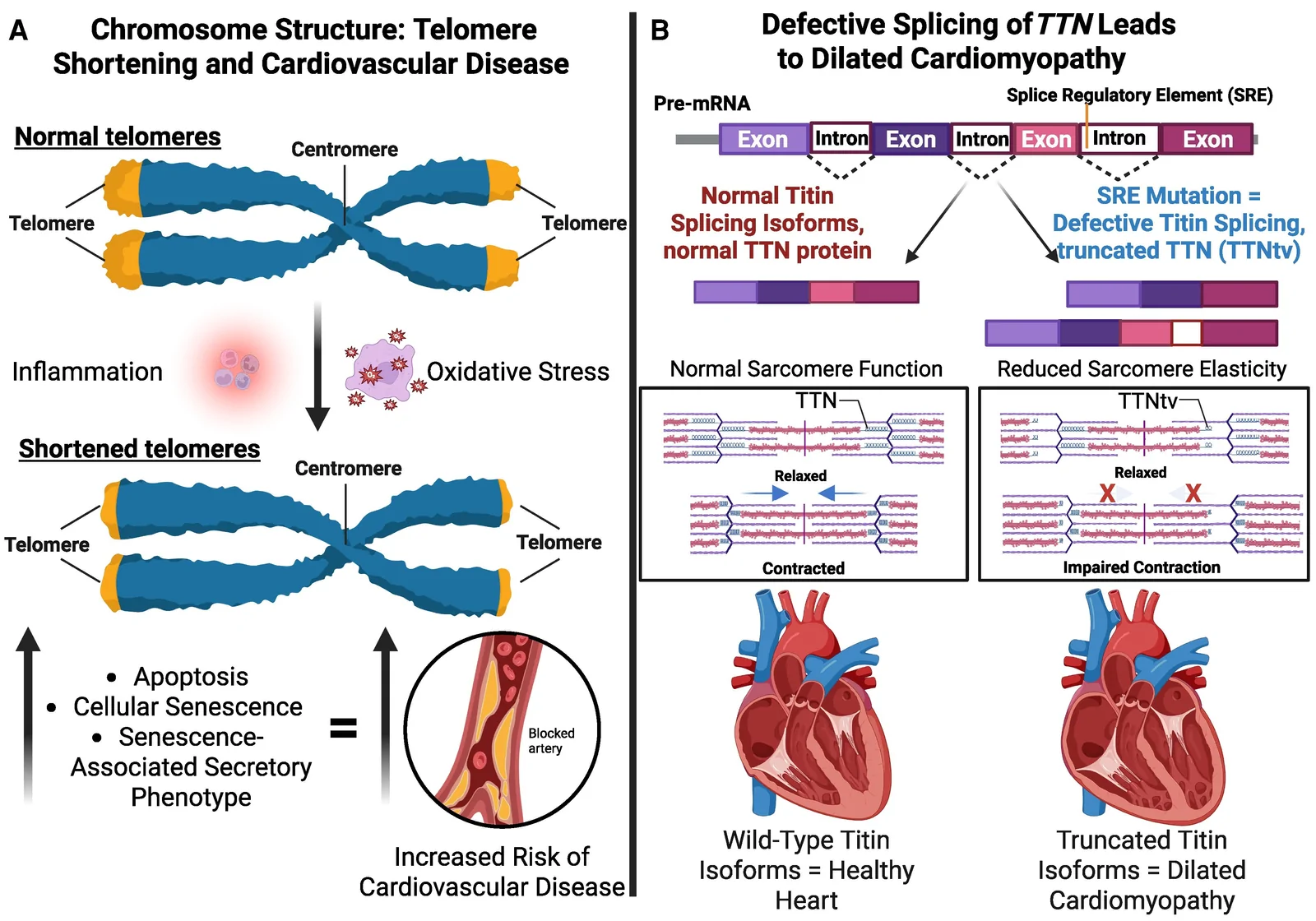
Regulatory element of the dark genome: chromosome structure and alternative splicing. (*A*) Overview of human chromosome structure, with key structural elements such as centromeres and telomeres annotated. Various factors such as age, oxidative stress, and chronic inflammation can result in progressive telomere shortening. Cells with shortened telomeres exit the cell cycle and enter senescence, which is associated with the secretion of a variety of molecules, collectively known as the senescence-associated secretory phenotype, and can become apoptotic. While conclusive studies remain to be published, some studies suggest telomere shortening may lead to an increased risk of cardiovascular disease.^16^ (*B*) Alternative splicing is a process by which different protein isoforms can be generated through the differential splicing of exons. This is governed, in part, by splice regulatory elements present within introns. Mutations in *TTN* introns have been shown to lead to splicing errors resulting in truncated Titin isoform expression.^17^ These can lead to reduced sarcomere elasticity and dilated cardiomyopathy.^18^

Other structural components of the dark genome, including centromeres, telomeres, satellite DNA, and tandem repeats, are now increasingly recognized as functionally important to cell function. Telomeres are repetitive DNA sequences at chromosome ends that protect chromosomes from degradation and fusion, and telomere shortening has well-established links to vascular ageing, cellular senescence, atherosclerosis, and cardiovascular risk (*Figure 3A*).^16,19^ In contrast, centromeres, which are specialized chromosomal regions required for accurate chromosome segregation during cell division, and satellite DNA, which consists of repetitive sequences found in centromeric and nearby pericentromeric regions, remain less well studied in cardiovascular disease.^20^ These regions are sources of active transcription rather than inert repeats.^20^ In cancer, e.g. dysregulated satellite transcription has been linked to chromatin instability, abnormal chromosome segregation, and stress responses,^21^ raising the possibility that similar mechanisms may also be relevant in cardiovascular pathology. This remains an important area for future investigation.

### RNA processing: alternative splicing

Alternative splicing joins different exon combinations from a single precursor RNA, allowing one gene to generate multiple transcripts and protein isoforms. This process is regulated in part by non-coding intronic sequences containing splicing-regulatory elements, which help control exon inclusion or exclusion and thereby shape isoform expression in human tissues.^22–24^ Mutations in intronic sequences can generate pathological splice variants and have been implicated in conditions including dilated cardiomyopathy,^18^ heart arrhythmia in myotonic dystrophy patients,^25^ hypertrophic cardiomyopathy,^26^ and hereditary long QT syndrome.^27^

This principle is illustrated by intronic variants in the *TTN* gene, which can disrupt healthy, physiological splicing, creating truncation variants of the Titin protein, causing reduced sarcomere elasticity and dilated cardiomyopathy (*Figure 3B*),^17^ as well as variants in *LMNA* and *MYBPC3*, which have been linked to aberrant splicing in cardiomyopathy and cardiac hypertrophy/heart failure.^28,29^ These examples highlight that the dark genome contributes to cardiovascular disease not only through regulatory DNA elements, but also through non-coding sequence motifs that govern RNA maturation.

### Transposable elements

Transposable elements represent the single largest category of genomic sequence in humans (∼45%).^30^ Transposable elements are mobile DNA sequences, mostly derived from ancient viral insertions or related to them, that can propagate throughout the genome via a ‘copy-and-paste’ mechanism, called retrotransposition. Through this action, they can influence gene regulation, chromatin structure, and genomic stability by introducing or disrupting regulatory elements.^31^ Transposable elements are broadly sub-classified based on the presence or absence of long terminal repeats (LTRs), which resemble retroviruses, and non-LTR elements. The latter includes long interspersed nuclear elements (LINEs), which are autonomous, and short interspersed nuclear elements (SINEs), such as Alu elements, which rely on LINE machinery for mobility.^32^

**Figure 4:**
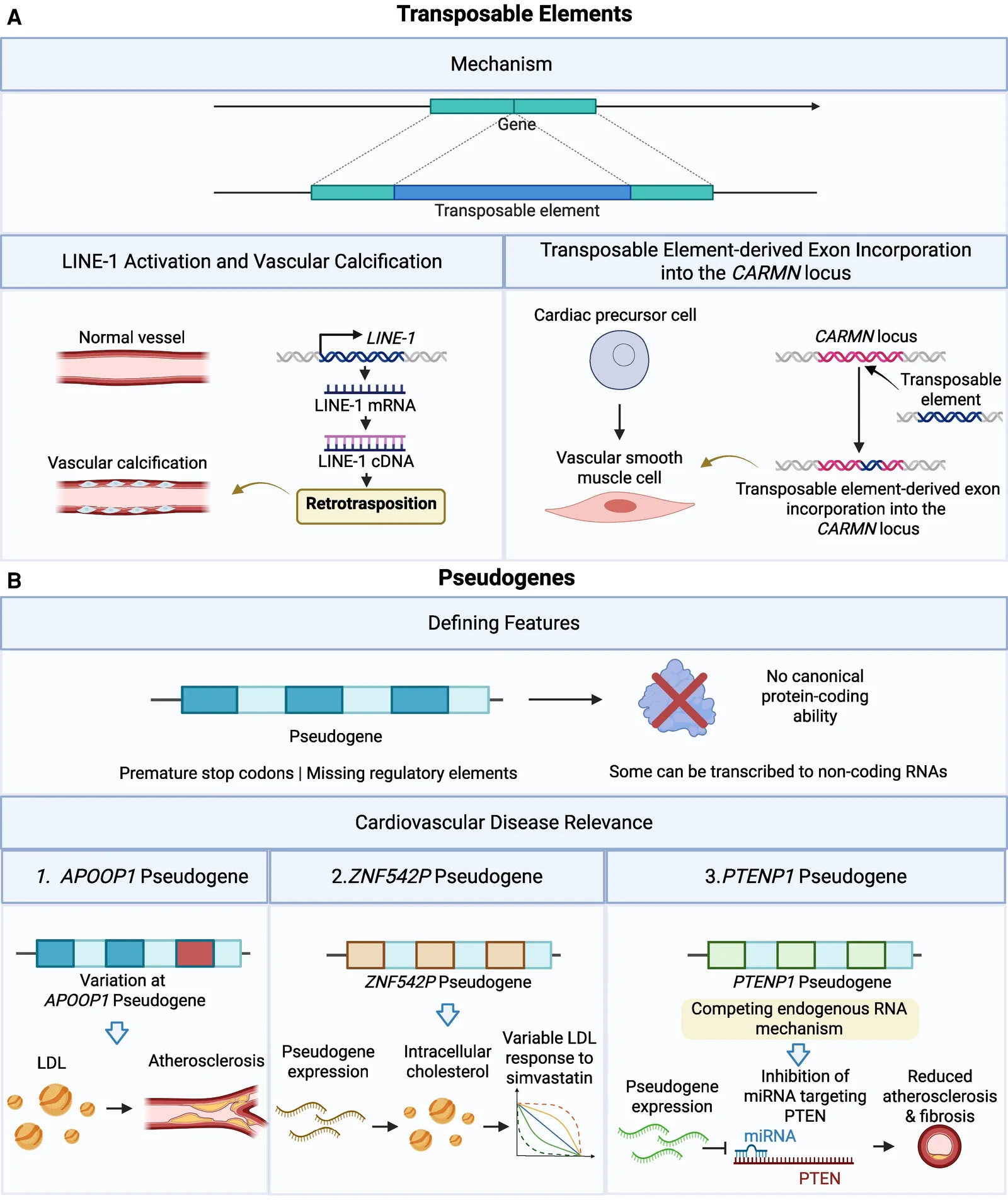
Transposable elements and pseudogenes as regulators of cardiovascular disease. (*A*) Transposable elements. Top, schematic overview showing how transposable element sequences can influence genomic organization and gene regulation. Bottom left, increased LINE-1 activation promotes transcription, cDNA generation, and retrotransposition, and has been linked to vascular calcification and vascular remodelling.^33^ Bottom right, transposable element-derived exon incorporation into the CARMN locus illustrates evolutionary co-option into a cardiovascular regulatory programme linked to differentiation from cardiac precursor cells to vascular smooth muscle cells.^34^ (*B*) Pseudogenes. Top: defining features of pseudogenes. Bottom: representative examples of cardiovascular relevance. Left: variation at the APOOP1 pseudogene locus has been associated with altered LDL cholesterol and atherosclerosis.^35^ Middle: ZNF542P expression has been linked to intracellular cholesterol levels and variable LDL-cholesterol response to simvastatin.^36^ Right: PTENP1 acts as a competing endogenous RNA that reduces miRNA-mediated repression of PTEN, thereby influencing pathways relevant to atherosclerosis and vascular fibrosis.^37^

The expression of most transposable elements is tightly constrained by multiple molecular processes that often differ between germline and somatic cells. Despite this high level of transcriptional control, transposable element expression and function are increasingly recognized in cardiovascular disease (*Figure 4A*). Vascular calcification is a key feature of atherosclerosis and is associated with common cardiovascular conditions. It has been shown that expression of the largest and most prevalent retrotransposon, LINE-1, is increased in calcified human arteries,^33^ and that silencing LINE-1 or inhibiting its activity can attenuate calcification and pro-inflammatory signalling.^33^ These results suggest that transposable element activation contributes to calcific pathology in cardiovascular disease. Transposable element-derived sequences can also be assimilated into cardiovascular gene networks; e.g. a transposable element-derived exon within the CARMN locus that contains miRNAs and a host gene long non-coding RNA (lncRNA), has been reported to contribute to differentiation of cardiac precursor cells into vascular smooth muscle cells.^34^ Increased expression of another class of transposable elements, endogenous retroviruses, has also been reported in patients with dilated cardiomyopathy.^38^ These observations suggest that transposable elements can influence cardiovascular biology both through pathological activation of transposition and through evolutionary incorporation into regulatory programmes.

### Pseudogenes

Pseudogenes are genomic sequences derived from ancient protein-coding genes that generally no longer encode functional proteins, most commonly due to disruptive mutations or loss of regulatory elements,^39^ and long considered non-functional relics.^40^ However, increasing evidence now shows that some pseudogenes are transcribed and can regulate gene expression in multiple disease contexts.^35,36^

Cardiovascular examples for functional pseudogenes are beginning to emerge (*Figure 4B*). In an Amish population, variation at the pseudogene *APOOP1* was associated with increased circulating low-density lipoprotein (LDL) cholesterol, and functional studies in zebrafish supported a role in hypercholesterolaemia and vascular plaque formation.^35^ In a separate study, expression of the pseudogene *ZNF542P* was associated with intracellular cholesterol levels and variability in LDL-cholesterol response to simvastatin, suggesting a role in treatment response.^36^ Another example is *PTENP1*, a pseudogene of *PTEN* gene, which has been implicated in smooth muscle cell proliferation,^37^ a process relevant to atherosclerosis^41^ and vascular fibrosis.^42^ Together, these observations suggest that pseudogenes may contribute to cardiovascular phenotypes through regulatory, rather than protein-coding, functions.

## Non-coding RNAs: essential regulators of cell function and dysfunction

Non-coding RNAs (ncRNAs) represent a major transcriptional output of the dark genome and an increasingly tractable regulatory layer that is now a major research focus. Significant advances in transcriptomic profiling and functional genomics have expanded ncRNA annotation across cardiovascular tissues and disease states, strengthening links between ncRNA regulation and cardiovascular phenotypes. Because many ncRNAs show marked cell type- and context-specific expression, they are attractive candidates for mechanistic investigation and therapeutic modulation. Together, these RNAs form a major interface through which the dark genome shapes gene-regulatory networks relevant to cardiovascular health and disease.

### Classes of non-coding RNAs and their genomic prevalence

**Figure 5:**
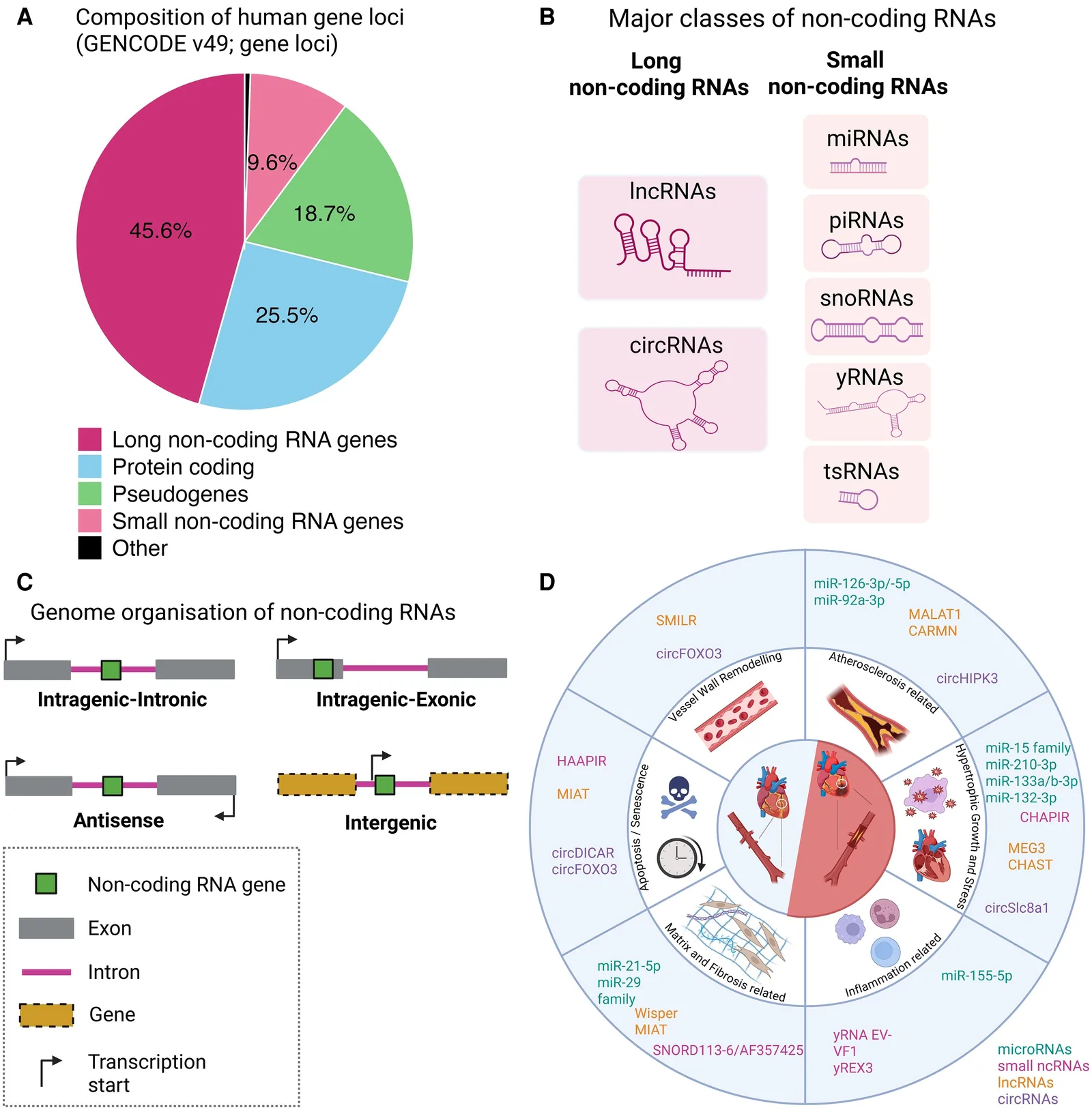
Genomic organization and structure of non-coding RNAs in the human genome. (*A*) Composition of human gene loci based on current GENCODE annotation (version 49). (*B*) Major classes of non-coding RNAs are broadly categorized into long non-coding RNAs (lncRNAs and circRNAs) and small non-coding RNAs (including miRNAs, snoRNAs, yRNAs, tsRNAs, and piRNAs). (*C*) Genomic organization of non-coding RNA loci, illustrating intergenic, intragenic (intronic and exonic), and antisense transcription relative to protein-coding genes. (*D*) Overview of key non-coding RNA classes and exemplars associated with major cardiovascular disease and disease-relevant processes, including atherosclerosis and cardiac diseases,^45–51^ cardiomyocyte hypertrophy and survival,^52–59^ vascular remodelling,^60–63^ apoptosis/senescence,^64–67^ inflammation,^68–71^ extracellular matrix regulation and fibrosis.^72–76^

Non-coding RNA classification usually combines transcript length with the biogenesis/processing to produce such transcripts. There are two major subtypes: small non-coding RNAs (typically <500 nucleotides) and long non-coding RNAs (>500 nucleotides).^43^ In the current GENCODE human genome annotation,^44^ 78 691 distinct genomic locations or ‘gene loci’ are catalogued. Of these, 20 097 are protein–coding, whereas the remainder comprise non-coding RNA genes (35 899 long non-coding RNA genes and 7563 small non-coding RNA genes), pseudogenes (14 701), and immunoglobulin/T-cell receptor gene segments (649) (*Figure 5A*).^44^ Genome annotation remains an active area, and continued improvements in sequencing technologies and functional genomics are expected to refine gene loci and non-coding RNA catalogues over time.^77^ Non-coding RNAs are broadly categorized into small non-coding RNAs, including miRNAs, piRNAs, snoRNAs, Y RNAs, and tsRNAs, and long non-coding RNAs, including lncRNAs and circRNAs, based on transcript length and biogenesis (*Figure 5B*). Genomically, non-coding RNA loci can be located between protein-coding genes (intergenic), within introns or exons of other genes (intragenic), or on the opposite strand to a protein-coding gene (antisense), each configuration with distinct implications for their regulation and function (*Figure 5C*). While the translational evidence remains variable across non-coding RNA classes, several have already been implicated in major cardiovascular disease processes (*Figure 5D*), and representative therapeutic strategies currently under preclinical or clinical evaluation are summarized in Supplementary data online, *Table S2*.

### Small non-coding RNAs in cardiovascular disease

Small non-coding RNAs comprise several classes, including miRNAs, small nucleolar RNAs (snoRNAs), small nuclear RNAs (snRNAs), tRNA-derived small RNAs (tsRNAs), and Y RNAs.

#### MicroRNAs in cardiovascular disease

**Figure 6:**
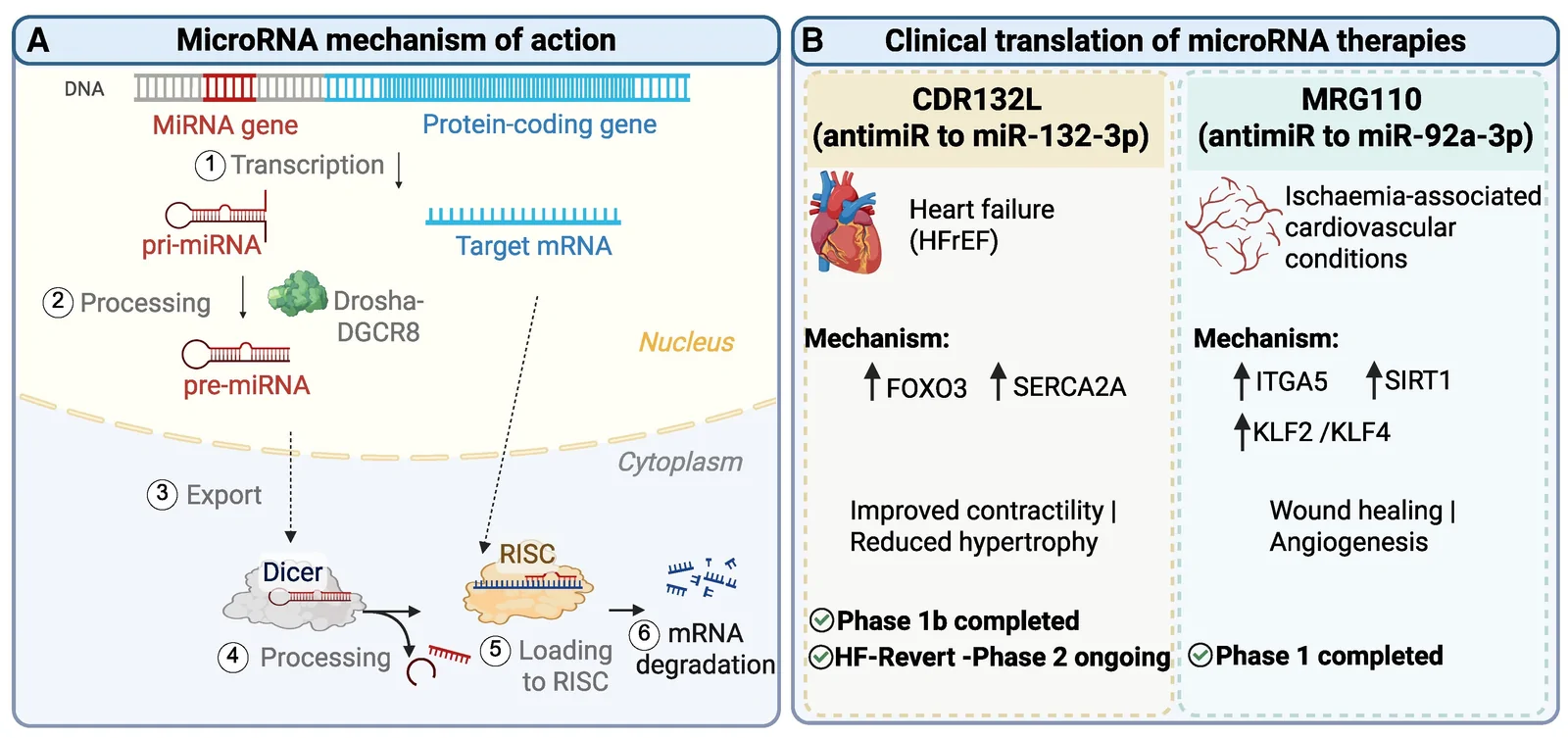
The role and mechanism of function of microRNAs in cardiovascular disease. (*A*) Canonical miRNA biogenesis and mechanism of action. (*B*) Clinical translation of miRNA-based therapies, illustrated by CDR132L (anti-miR-132-3p) for heart failure with reduced ejection fraction^81,82^ and MRG110 (anti-miR-92a-3p) for ischaemia-associated angiogenic indications.^83^

MiRNAs are short (∼22-nucleotide) RNAs that bind to target messenger RNAs and typically repress gene expression by reducing their stability and translation.^78^ Their biogenesis, expression patterns, and cardiovascular roles have been comprehensively reviewed elsewhere^79,80^; an overview of canonical miRNA biogenesis and therapeutic modalities is summarized in *Figure 6*. Although several miRNAs have shown promise in preclinical cardiovascular studies, few have advanced to clinical trials (see Supplementary data online, *Table S2*). Here we briefly focus on the most clinically advanced examples, and readers are referred to a recent comprehensive review in the field for in-depth detail of miRNAs in cardiovascular research.^84^

Modulation of miRNA function has progressed through two main strategies: inhibition using antisense oligonucleotides (ASOs, anti-miRs) and restoration using synthetic mimics. As an example, CDR132L is an anti-miR targeting miR-132-3p, which acts by de-repressing FOXO3 and SERCA2A, promoting improved contractility and reduced cardiomyocyte hypertrophy (*Figure 6B*).^52,81^ In a Phase Ib trial, CDR132L demonstrated good tolerability and a median 23.3% N-terminal pro-B-type natriuretic peptide (NT-proBNP) reduction,^82^ and is currently under evaluation in the HF-REVERT trial (NCT05350969) in patients with reduced ejection fraction after myocardial infarction.^81^ MRG110 targets the anti-angiogenic miR-92a-3p, de-repressing targets including ITGA5, SIRT1, and KLF2/KLF4 to promote angiogenesis and wound healing (*Figure 6B*).^83^ A first-in-human study demonstrated sustained reduction of circulating miR-92a-3p,^83^ with a further Phase I trial ongoing (NCT03494712); given that miR-92a-3p inhibition reduces atherosclerosis in preclinical models,^85^ these results are of cardiovascular interest. MiRNA mimics offer a complementary approach to restore miRNA activity. Unbiased mimic screens have identified miRNAs capable of promoting cardiac regeneration,^86^ and mimic delivery has shown cardioprotective effects in preclinical ischaemia models.^87^ Emerging delivery strategies using lipid nanoparticle and modified RNA-based approaches are resolving delivery challenges, yet this is an area of key focus to elevate safety and efficacy.^88,89^ These early-phase studies support the feasibility of miRNA-targeted therapeutics, though definitive evaluation of clinical benefit will require adequately powered cardiovascular trials.

#### Beyond microRNAs: emerging roles for SnoRNAs, Y RNA fragments, TsRNAs, and PiRNAs

**Figure 7:**
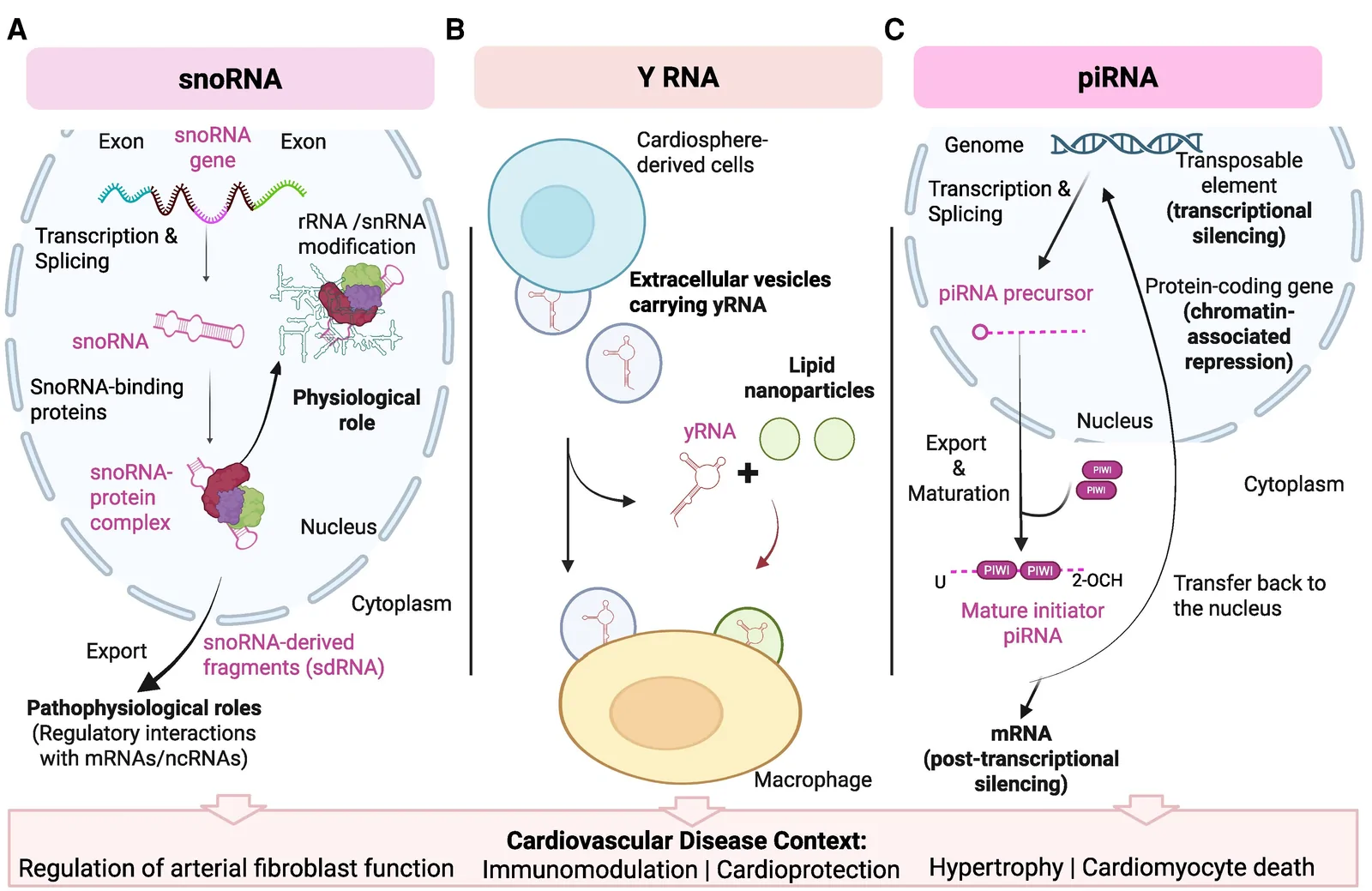
Mechanisms of action of selected small non-coding RNAs in cardiovascular biology. (*A*) Example of small nucleolar RNA (snoRNA) mechanism of function. SnoRNAs guide chemical modification of ribosomal and small nuclear RNAs (snRNAs) within snoRNA–protein complexes and can generate snoRNA-derived fragments with regulatory functions in disease contexts. (*B*) Example of y RNA mechanism of action. Y RNAs can be packaged within extracellular vesicles (EVs) and transferred between cells, modulating immune responses and cardioprotection following injury. The figure also represents an alternative therapeutic modality in which yRNAs can be loaded into lipid nanoparticles and delivered independently to EVs.^68,69^  **C.** Example of PIWI-interacting RNA (piRNA) mechanism of function. PiRNAs bind PIWI proteins to regulate gene expression through post-transcriptional silencing and chromatin-associated repression, with emerging roles in cardiomyocyte hypertrophy and survival

Although miRNAs are the best characterized small non-coding RNAs, evidence indicates that other small non-coding RNA classes contribute to cardiovascular phenotypes. A greater understanding of these is crucial to reveal novel pathways and to identify therapeutic targets. Other types of small non-coding RNAs, include snoRNAs, Y RNA-derived fragments, tsRNAs, and piRNAs (see Supplementary data online, *Table S2*, *Figure 7*). Evidence for these in cardiovascular disease is less mature; however, for some classes, including snoRNAs, mechanistic links are emerging. SnoRNAs guide the modification of other RNAs (most commonly ribosomal RNAs and snRNAs) (*Figure 7A*). In the context of vascular remodelling, snoRNAs, including SNORD113-6/AF357425 have been shown to modulate signalling pathways which are important for arterial fibroblast function.^72^ In parallel, broader human studies report co-ordinated regulation of multiple snoRNAs in diseased vascular tissue, supporting a wider contribution to cardiovascular pathology.^90^

Y-RNA fragments are another class of small non-coding RNAs. Although their functions were unclear for years, they have been linked to RNA quality control, proliferation, and DNA replication (*Figure 7B*).^91^ In the context of cardiac injury, Marbán and colleagues identified a short Y RNA fragment (EV-YF1) within extracellular vesicles (EVs) from cardiac cells.^68^ EVs are small particles released by cells that carry biological cargo, such as small non-coding RNAs, and their composition can change in disease. In ischaemia–reperfusion models, EVs containing EV-YF1 were reported to signal to immune cells and increase the expression of anti-inflammatory cytokines, conferring cardioprotection.^68^ Importantly, this observation has suggested a novel therapeutic approach: synthetic Y RNAs can be incorporated within lipid nanoparticles, enabling mass production of advanced therapeutics for cardiovascular disease.^69^ Recent work has extended this paradigm by identifying additional EV-enriched Y RNA species with therapeutic potential, including yREX3, which exerts cardioprotection.^69^

Another class of small ncRNAs, tsRNAs are generated from transfer RNAs (tRNAs) that normally deliver amino acids to the ribosome during protein synthesis.^92^ TsRNAs are increasingly recognized as stress-responsive regulators in the cardiovascular system, with multiple fragments detected in models of cardiac hypertrophy, ischaemia–reperfusion injury, pulmonary vascular disease, and mitochondrial cardiomyopathy.^93^ However, functional studies remain relatively limited and confined to experimental settings.

Finally, piRNAs are a class of small RNAs that bind PIWI proteins (Argonaute-family RNA-binding proteins that partner with small RNAs) to regulate gene expression (*Figure 7C*). They are mainly found in reproductive tissues and are best known for silencing transposable elements, but can also affect RNA stability and gene regulation at the chromatin level.^94^ In cardiovascular disease, a small number of studies have suggested roles for specific cardiac piRNAs, including CHAPIR (linked to pathological hypertrophy)^53^ and HAAPIR (linked to cardiomyocyte apoptosis).^64^ However, the evidence remains limited.

More broadly, other small non-coding RNA species, including vault RNAs,^95^ 7SL/SRP RNA-derived fragments^96^ and 7SK RNA,^97^ as well as rRNA-derived fragments, remain poorly characterized in cardiovascular health and disease. Improved annotation, cell type-resolved profiling, and functional perturbation studies are therefore needed to define which of these RNAs are causal drivers, biomarkers, or therapeutic targets in cardiovascular disease. Such efforts have the potential to expand the repertoire of regulatory pathways implicated in cardiovascular disease and to identify new candidates for biomarker development and therapeutic modulation.

### Long non-coding RNAs in cardiovascular disease

**Figure 8:**
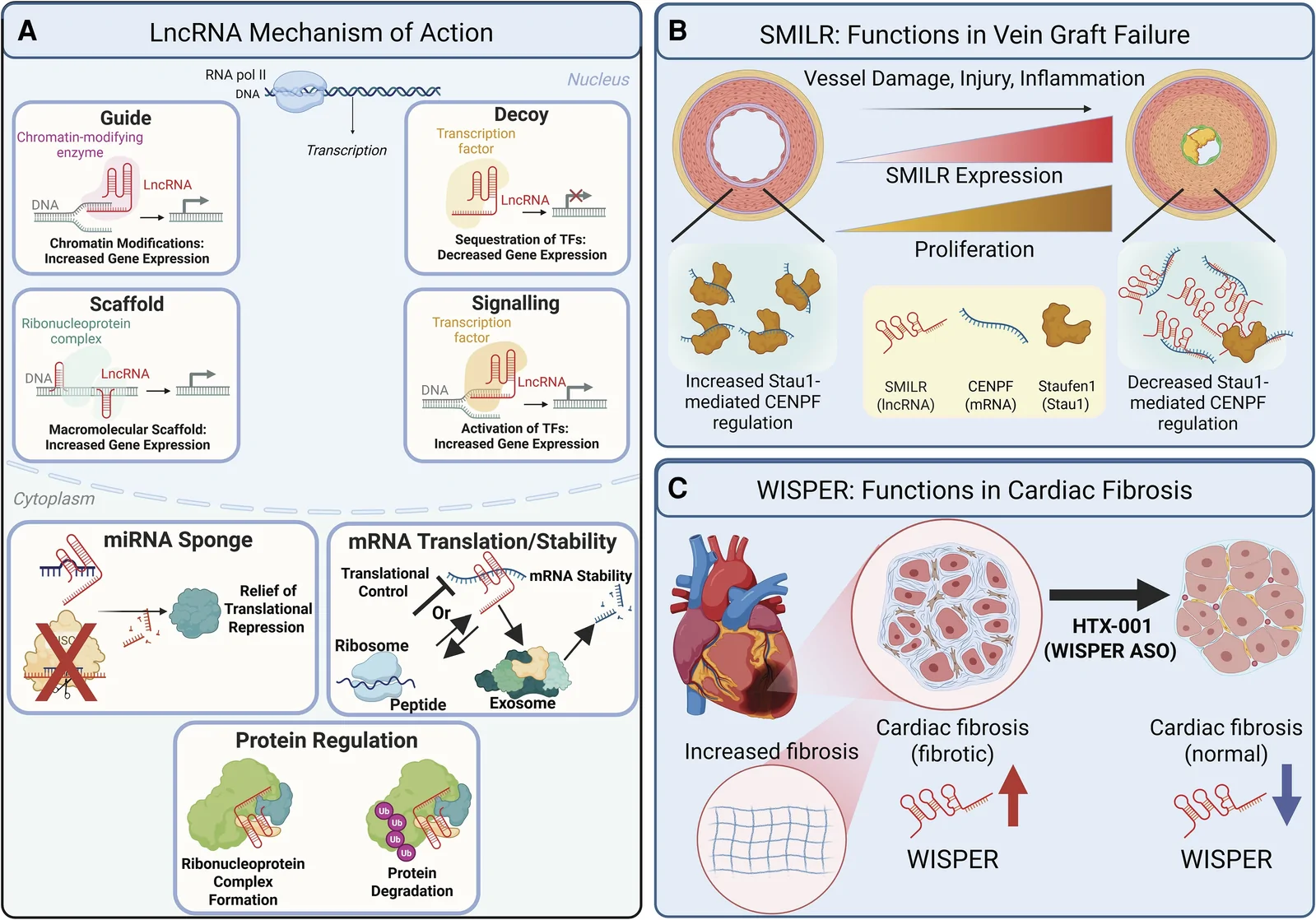
Regulatory mechanisms of long non-coding RNAs in cardiovascular disease. (*A*) Canonical Long non-coding RNA (lncRNA) biogenesis and mechanism of action. (*B*) The lncRNA SMILR becomes expressed in vascular smooth muscle cells (vSMCs) in response to vascular damage, pro-inflammatory stimuli, or growth factors. SMILR binds to the mRNA of the mitotic gene CENPF, where it acts to shield CENPF from Stauffen1 (Stau1)-mediated degradation, thereby promoting vSMC proliferation.^60–62^ (C) The lncRNA WISPER is upregulated in cardiac fibroblasts from infarcted hearts and promotes cardiac fibrosis and is very selective for cardiac fibroblasts. An antisense oligonucleotide (ASO), HTX-001, decreases WISPER levels, reduces cardiac fibrosis, and improves cardiac function.^73^

Whereas small non-coding RNAs primarily act through post-transcriptional mechanisms, long non-coding RNAs add an additional regulatory layer that can shape transcriptional programmes, chromatin state, and cell phenotype (*Figure 8A*). The long non-coding RNA category comprises long non-coding RNAs (lncRNAs) and circular RNAs (circRNAs), two abundant classes with recognized roles in cardiovascular health and disease (see Supplementary data online, *Table S2*).^43,98–100^ Although the functions of cardiovascular lncRNAs have been comprehensively reviewed elsewhere,^101,102^ here we highlight several well-defined lncRNAs and briefly discuss how these contribute to cardiovascular disease-relevant pathways (*Figure 8B* and *C*). In vascular smooth muscle cells (vSMCs), CARMN promotes a contractile identity and stabilizes the vSMC phenotype,^45^ while the lncRNA SMILR becomes expressed in response to proliferative stimuli preferentially in vSMCs, where it acts to accelerate cell proliferation, a pathogenic phenotype of several cardiovascular diseases (*Figure 8B*).^60–62^ LncRNAs involved in matrix remodelling, such as the cardiac fibroblast-enriched Wisper, orchestrate myofibroblast activation and extracellular matrix deposition (*Figure 8C*).^73^ Together, these representative lncRNAs highlight how long non-coding transcription integrates multiple regulatory modalities to shape cardiovascular disease progression.

#### Therapeutic targeting of lncRNAs

Substantial experimental evidence now demonstrates that blocking or enhancing the activity of lncRNAs may offer therapeutic benefit across many cardiovascular pathologies. Short interfering RNA (siRNA) and short hairpin RNA (shRNA) have been developed to reduce gene function primarily based on the naturally occurring mechanisms of RNA interference (RNAi), and these same technologies are now also being applied to target lncRNAs. A discussion of the mechanisms of RNAi is beyond the scope of this review and has been reviewed comprehensively.^103^ There is currently a scarcity of lncRNA-targeting siRNA in clinical studies. This is partly due to the relatively recent appreciation of lncRNAs and their role in disease, and due to poor cross-species conservation compared to protein-coding genes, which impedes pre-clinical studies in animal models. Despite these challenges, a greater cell-specific expression pattern of lncRNAs makes them attractive therapeutic targets.

#### Circular RNAs in cardiovascular disease

**Figure 9:**
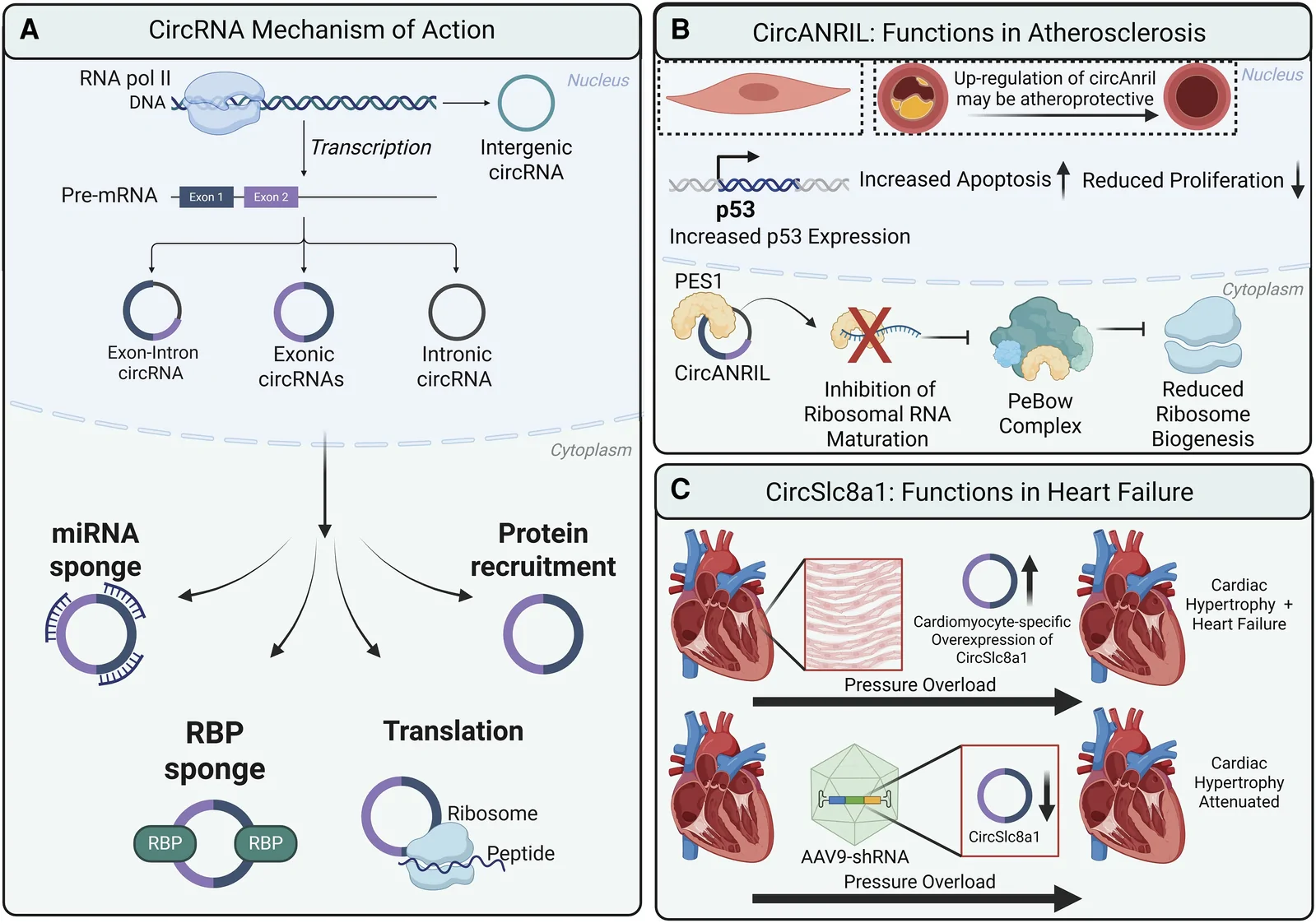
Regulatory mechanisms of circular RNAs in cardiovascular disease. (*A*) Canonical circular RNA (circRNA) biogenesis and mechanisms of action, including acting as microRNA (miRNA) sponge, RNA binding protein (RBP) sponge, and roles in translation process and protein recruitment. (*B*) CircANRIL is reported to bind to PES1, a component of the PeBow complex, essential for ribosome biogenesis. By binding to PES1, circANRIL prevents ribosomal RNA (rRNA) processing, leading to reduced ribosome biogenesis. Reduced ribosome biogenesis triggers the increased expression of p53, a master regulator of the DNA damage and apoptosis response. This leads to increased apoptosis and reduced proliferation in vascular smooth muscle cells (vSMCs).^104^  **C.** Overexpression of the circRNA CircSlc8a1 accelerates cardiac hypertrophy and heart failure in a pressure overload heart model. Adeno-associated virus (AAV9)-mediated RNAi knockdown of CircSlc8a1 using a short hairpin RNA (shRNA) successfully attenuates cardiac hypertrophy.^54^

Circular RNAs (circRNAs) are covalently closed, single-stranded RNAs that can be generated from both protein-coding and non-coding genes and form stable RNA circles. CircRNAs have been implicated in regulatory roles, including interaction with RNA-binding proteins, modulation of transcription, and, in some cases, regulation of miRNA activity (*Figure 9A*). Their stability and tissue-specific expression patterns have made them attractive candidates as cardiovascular biomarkers and potential therapeutic targets.^105^

Within cardiomyocytes, several circRNAs have been shown to influence stress adaptation and pathological growth. CircRNAs participate in metabolic and inflammatory injury. For example, reduced expression of circRNA DICAR drives cardiomyocyte pyroptosis in diabetic cardiomyopathy,^65^ and vascular circRNAs such as circANRIL shape proliferative and inflammatory responses linked to atherosclerosis (*Figure 9B*).^104^ CircSlc8a1 modulates hypertrophic signalling (*Figure 9C*)^54^ while circFOXO3 promotes cellular senescence through interaction with proteins that normally restrain stress responses and senescence pathways.^66^ CircRNAs also operate in the vascular compartment, where endothelial transcripts such as circHIPK3 support angiogenic competence and vascular repair.^106^ Together, these mechanistically defined examples illustrate how circular transcript architecture enables diverse regulatory roles across cardiac and vascular compartments.

### Network-level regulation by non-coding RNAs in cardiovascular disease

Non-coding RNAs act within interconnected regulatory networks in which multiple RNA classes converge to shape gene-expression programmes relevant to cardiovascular disease. Rather than functioning as isolated regulators, miRNAs, lncRNAs, and circRNAs frequently participate in shared circuits, including competing endogenous RNA networks in which lncRNAs or circRNAs modulate miRNA availability and thereby influence downstream mRNA targets, though the mechanistic underpinnings of such networks that can account for the relative stoichiometries of messenger RNAs and lncRNAs remain unclear.^79,107^ Such lncRNA–miRNA–mRNA and circRNA–miRNA–mRNA interactions have been described in myocardial infarction, heart failure, and atherosclerosis, where they affect processes such as cardiomyocyte survival, fibroblast activation, and vascular inflammation.^108^

Beyond these networks, snoRNA-derived fragments and tRNA-derived small RNAs also intersect with miRNA- and lncRNA-centred regulatory modules,^109–112^ although the overall prevalence and impact of such interactions in cardiovascular disease remain poorly characterized.^113^ At the tissue level, EVs provide a further layer of integration by packaging distinct non-coding RNA species, enabling co-ordinated transfer of regulatory signals between cardiomyocytes, fibroblasts, endothelial cells, and immune cells during injury and remodelling.^114^ Together, these observations support a view of the cardiovascular dark genome as a distributed complex regulatory network in which multiple non-coding RNA classes intersect to orchestrate cardiovascular disease-relevant transcriptional programmes. Defining this network will require sustained research that integrates perturbation experiments with bioinformatics approaches with the aim of identifying causal regulators that could be prioritized as therapeutic targets for cardiovascular disease.^115,116^

### Will there be other non-coding RNAs that emerge?

Despite rapid progress in annotation, a subset of non-coding transcripts remains difficult to classify. One example is non-coding RNAs derived from 3′ untranslated regions (3′ UTRs), which can exhibit subcellular localizations distinct from their parent messenger RNAs,^117,118^ and may therefore have distinct regulatory functions.^119^ However, their biogenesis, mechanism, and regulation are still largely unknown.^120^ Ongoing advances in transcriptome profiling and RNA-focused functional genomics are now enabling more systematic research of such atypical RNA species. Whether these non-canonical RNAs contribute to cardiovascular physiology or disease remains to be determined.

## Tools, technologies, and methodological challenges in dark genome research

**Figure 10:**
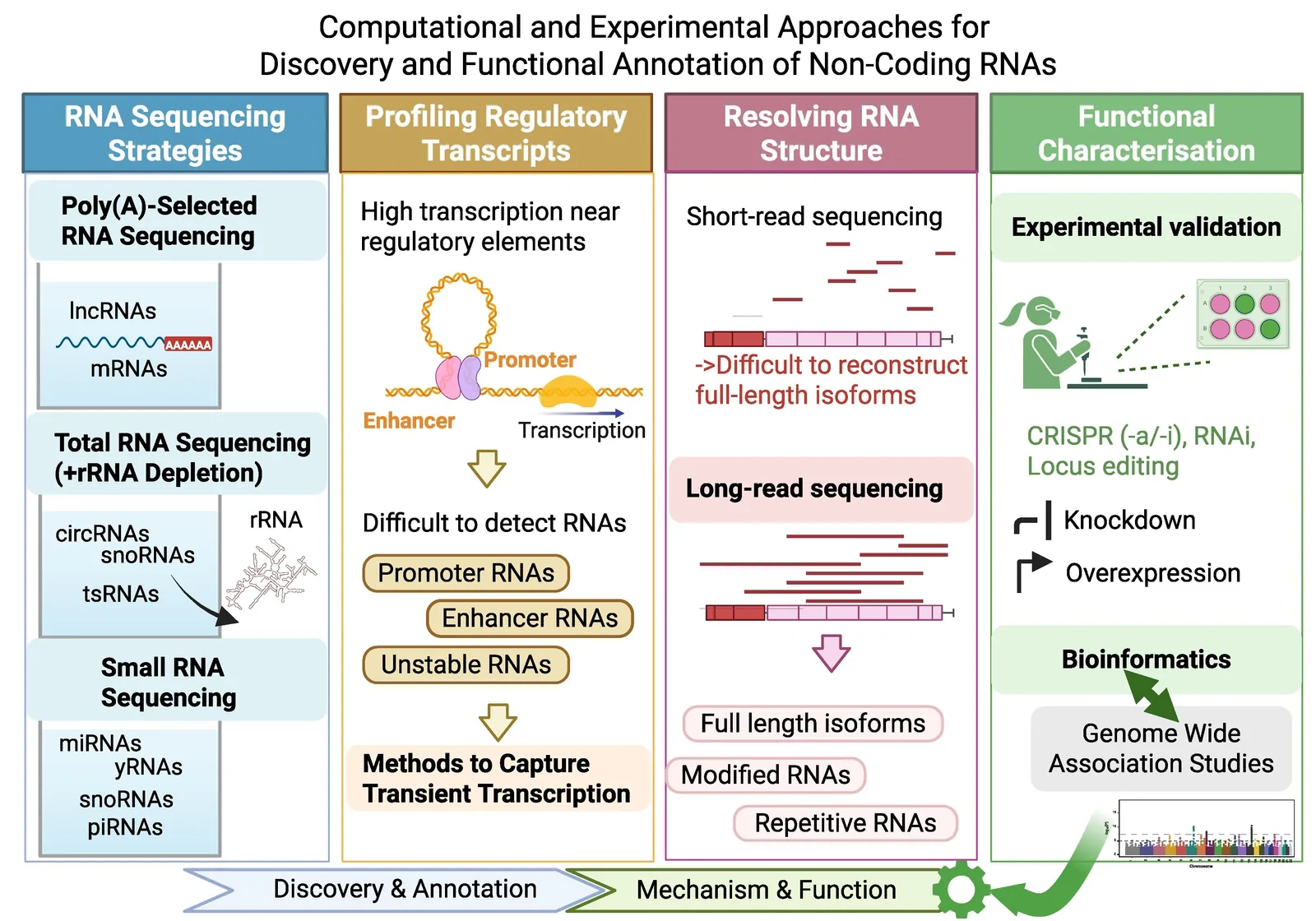
Computational and experimental approaches for discovery and functional annotation of non-coding RNAs. Overview of key technologies used to identify and characterize non-coding RNAs, including RNA sequencing strategies (poly(A)-selected, total RNA and small RNA sequencing), methods to capture transient regulatory transcription, short- and long-read sequencing for isoform resolution, and approaches for functional perturbation: RNA interference (RNAi), CRISPR-based modulation and locus editing

A range of computational approaches is now enabling discovery, functional annotation, and therapeutic targeting of dark genome elements across cardiovascular tissues and disease context. While methods for regulatory DNA elements have been discussed in Section 2.2, here we focus on approaches for non-coding RNA discovery and functional characterization (*Figure 10*), before addressing broader methodological challenges that apply across dark genome research.

### RNA sequencing strategies for non-coding RNA discovery

High-throughput RNA sequencing profiles RNA species and their abundance across tissues, cell types, disease states, or in response to stimuli. The discovery and characterization of non-coding RNAs (ncRNAs) rely on high-throughput sequencing strategies tailored to specific RNA populations. While standard poly(A)-selected RNA-sequencing captures many lncRNAs, total RNA library preparations utilizing ribosomal RNA depletion are necessary to identify circular and non-polyadenylated transcripts. Small ncRNAs, such as miRNAs, tRNA fragments, and Y RNA fragments, often require dedicated size-selection workflows. Additionally, short-read sequencing often struggles with complex or repetitive isoforms; however, long-read sequencing and targeted capture can provide more accurate full-length transcript reconstruction.^121,122^

Interpretation requires care, as library preparation can introduce sequence- and modification-dependent biases (notably for tRNA-derived fragments), and complementary validation is often needed.^123^ Moreover, a substantial fraction of transcription occurs near regulatory regions such as promoters and enhancers, producing RNAs that are often unstable and therefore difficult to detect in steady-state RNA sequencing. Methods that profile nascent RNA can reveal this transient transcriptional activity and help map where transcription initiates and how it is regulated.^124,125^

### Decoding non-coding function: perturbation readouts and AI-guided prediction

Beyond identification, establishing function of RNA species necessitates integrating perturbation assays, such as CRISPR-based repression or activation and quantitative readouts like nascent RNA profiling and chromatin profiling, which captures transient transcriptional activity at enhancers and promoters. To manage the scale of these data, machine learning and AI-guided frameworks are employed to prioritize candidate regulatory elements and translate non-coding genetic risk into causal cardiovascular mechanisms.^126,127^

### Methodological challenges

Despite rapid progress, several challenges limit the reliable identification and functional characterization of dark genome elements in cardiovascular research. First, the lack of standardized protocols and analytical pipelines across studies makes direct comparison of results difficult, and community-agreed minimum reporting standards are needed to improve reproducibility.^128^ Second, current genome annotations remain incomplete as functional evidence exists for only a small fraction of the tens of thousands of annotated non-coding loci, while many genuinely functional elements likely remain unannotated.^77^ Third, because the heart and vasculature contain multiple distinct cell types, studies using bulk tissue average signals across cardiomyocytes, fibroblasts, endothelial cells, and immune cells, potentially masking cell-type-specific regulatory activity.^129^ Single-cell and single-nucleus approaches are resolving this heterogeneity; however, most widely used single-cell transcriptomic datasets still rely on poly(A)-based workflows and therefore provide incomplete coverage of many non-coding RNA classes, although more recent total-RNA methods are starting to address this limitation.^130^ Fourth, demonstrating that a non-coding locus is functionally important, rather than merely associated or transcribed, remains technically demanding. For many annotated non-coding loci implicated in cardiovascular disease, causal roles, target genes, and underlying mechanisms have yet to be established.^131^ Fifth, many dark genome elements, particularly lncRNAs, show limited conservation between humans and the animal models used in preclinical research, complicating experimental validation and translation.^132^ Finally, the majority of genetic variants associated with cardiovascular disease in large population studies map to non-coding regions, yet linking these variants to specific regulatory mechanisms and target genes remains a major unsolved challenge.^133^

### Current limitations and next steps

Three priorities stand out for the field. First, expanded profiling of cardiovascular cell types using long-read and single-cell sequencing will broaden the catalogue of functionally relevant dark genome elements. Second, scalable experimental tools, including CRISPR-based screens^115^ across cardiovascular cell types, are needed to systematically identify which dark genome elements are causally important, and improved computational methods will help integrate these findings with existing genomic and clinical data. Third, translating dark genome discoveries into clinical benefit will require both advances in targeted delivery of nucleic acid therapeutics^134^ and integration of non-coding regulatory information into risk prediction frameworks.^135^ Addressing these challenges will ultimately determine whether the dark genome can be fully exploited for cardiovascular diagnosis and therapy.

## Emerging concepts in dark genome biology

The dark genome is increasingly recognized as a source of functional outputs beyond canonical RNAs. In addition to regulatory RNAs, some loci generate small bioactive peptides, and others appear to act through multiple co-existing mechanisms at the DNA, RNA, and peptide levels. In this section, we highlight these emerging concepts and their relevance to cardiovascular biology.

### The dark proteome and small open Reading frames

Advances in transcriptomics, ribosome profiling, and proteogenomics have revealed that some loci previously annotated as non-coding generate functional micropeptides from small open reading frames, indicating that part of the dark genome encodes peptides that were historically excluded from genome annotations. These micropeptides are typically <100 codons in length but can exert meaningful regulatory effects, influencing cellular metabolism, stress responses, and calcium handling.^136^

While these micropeptides remain poorly annotated, several have already been shown to exert direct, mechanistically defined effects in cardiovascular tissues. Micropeptides such as DWORF,^137^ and MOXI/mitoregulin^138^ have demonstrated roles in modulating cardiomyocyte contractility, mitochondrial function, and metabolic adaptation, while others, such as SPAAR, influence endothelial signalling and angiogenic responses.^139^ While micropeptide function is better defined in cardiomyocytes and endothelial cells, emerging data now implicate micropeptides in fibroblast activation and vascular smooth muscle phenotypic switching; however, these roles are supported by a relatively small number of recent studies and remain to be explored in depth.^140,141^ Collectively, these discoveries illustrate that the dark genome contributes not only regulatory RNAs but also peptide-coding potential that broadens the scope of gene expression in cardiovascular biology.

### Multifunctional genetic loci

Since the discovery of the first non-coding genes, it was acknowledged that some regions of the dark genome can exert regulatory effects through multiple, co-existing modalities, challenging the traditional separation of coding, non-coding, and regulatory DNA. At such loci, the DNA sequence can function as an enhancer or chromatin regulatory element, while the ‘non-coding’ RNA transcript mediates additional effects through transcriptional or post-transcriptional mechanisms.^142^ In some cases, as discussed in the previous section, these same transcripts also harbour small open reading frames that encode biologically active micropeptides, creating multimodal loci in which RNA and peptide outputs can mediate distinct, and in some contexts divergent, regulatory effects.

The LINC00961/SPAAR locus exemplifies RNA-peptide multimodality, as the non-coding transcript and the encoded micropeptide exert distinct and, in angiogenic assays, opposing effects on endothelial behaviour.^139^ A further exemplar is the chromosome 9p21.3 coronary artery disease risk locus, where a non-coding, enhancer-rich interval contains risk alleles that alter inflammatory transcription factor binding and cis-regulatory activity, while the same region also produces ANRIL isoforms with demonstrable effects on vascular cell phenotypes (including circANRIL effects on ribosomal RNA maturation and cell proliferation and apoptosis), illustrating how a single locus can integrate DNA-level regulatory and RNA-mediated mechanisms.^143^ More broadly, cardiac enhancer-associated lncRNA loci illustrate how a single genomic region can integrate DNA- and RNA-based regulatory functions, underscoring the multimodal nature of dark-genome regulation in cardiovascular biology.^73^ Taken together, these examples highlight that cardiovascular risk loci and disease-associated transcripts may encode overlapping DNA-, RNA-, and peptide-level functions, and that a locus-centred view is often necessary to define causal mechanisms and prioritize translational targets.

## Conclusions

The study of the dark genome has now reached a critical juncture. Two decades of rapid advances in sequencing technologies have extensively characterized the different DNA and RNA elements that our genome can produce. In addition to the discussed emerging concepts, key challenges remain. While progress has been made to solve long-standing DNA sequencing challenges, including the complete sequencing of the human genome (telomere-to-telomere),^144^ understanding the dynamic interplay between the various elements comprising this sequence remains an enormous challenge. This includes understanding how the 3D spatial architecture is governed through thousands of chromatin loops, how this varies between cell types, and if and how this architecture changes in response to disease or stimuli,^145^ and understanding how specific enhancers shape gene expression patterns hundreds of thousands of base pairs away. Understanding these processes will clarify our understanding of the dark genome. Clinical translation of key pre-clinical findings is also a critical next step. As discussed, a number of non-coding elements emerge now as either targets or therapeutic entities in various phases of clinical studies in cardiovascular medicine. The number of clinically evaluated non-coding elements is expected to increase substantially as these elements become fully functionally characterized.

## Supplementary Material

ehag514_Supplementary_Data

## Declarations

### Data Availability

No data were generated or analysed for or in support of this paper.

## References

[1] Musolino PL, Rosser SJ, Brittan M, Newby DE, Berry C, Riley PR, et al Gene therapy in cardiac and vascular diseases: a review of approaches to treat genetic and common cardiovascular diseases with novel gene-based therapeutics. Cardiovasc Res 2025;121:1843–55. 10.1093/cvr/cvaf109 40853254 PMC12551389

[2] ENCODE Project Consortium . An integrated encyclopedia of DNA elements in the human genome. Nature 2012;489:57–74. 10.1038/nature11247 22955616 PMC3439153

[3] Leypold NA, Speicher MR. Evolutionary conservation in noncoding genomic regions. Trends Genet 2021;37:903–18. 10.1016/j.tig.2021.06.007 34238591

[4] Huttenhofer A, Schattner P, Polacek N. Non-coding RNAs: hope or hype? Trends Genet 2005;21:289–97. 10.1016/j.tig.2005.03.007 15851066

[5] Carninci P, Kasukawa T, Katayama S, Gough J, Frith MC, Maeda N, et al The transcriptional landscape of the mammalian genome. Science 2005;309:1559–63. 10.1126/science.1112014 16141072

[6] Willingham AT, Gingeras TR. TUF love for “junk” DNA. Cell 2006;125:1215–20. 10.1016/j.cell.2006.06.009 16814704

[7] Destici E, Zhu F, Tran S, Preissl S, Farah EN, Zhang Y, et al Human-gained heart enhancers are associated with species-specific cardiac attributes. Nat Cardiovasc Res 2022;1:830–43. 10.1038/s44161-022-00124-7 36817700 PMC9937543

[8] Deviatiiarov RM, Gams A, Kulakovskiy IV, Buyan A, Meshcheryakov G, Syunyaev R, et al An atlas of transcribed human cardiac promoters and enhancers reveals an important role of regulatory elements in heart failure. Nat Cardiovasc Res 2023;2:58–75. 10.1038/s44161-022-00182-x 39196209

[9] Gacita AM, Dellefave-Castillo L, Page PGT, Barefield DY, Wasserstrom JA, Puckelwartz MJ, et al Altered enhancer and promoter usage leads to differential gene expression in the normal and failed human heart. Circ Heart Fail 2020;13:e006926. 10.1161/CIRCHEARTFAILURE.120.006926 32993371 PMC7577963

[10] Bertero A, Rosa-Garrido M. Three-dimensional chromatin organization in cardiac development and disease. J Mol Cell Cardiol 2021;151:89–105. 10.1016/j.yjmcc.2020.11.008 33242466 PMC11056610

[11] Gacita AM, Fullenkamp DE, Ohiri J, Pottinger T, Puckelwartz MJ, Nobrega MA, et al Genetic variation in enhancers modifies cardiomyopathy gene expression and progression. Circulation 2021;143:1302–16. 10.1161/CIRCULATIONAHA.120.050432 33478249 PMC8009836

[12] Grandi FC, Modi H, Kampman L, Corces MR. Chromatin accessibility profiling by ATAC-seq. Nat Protoc 2022;17:1518–52. 10.1038/s41596-022-00692-9 35478247 PMC9189070

[13] Johnson DS, Mortazavi A, Myers RM, Wold B. Genome-wide mapping of in vivo protein-DNA interactions. Science 2007;316:1497–502. 10.1126/science.1141319 17540862

[14] Shiraki T, Kondo S, Katayama S, Waki K, Kasukawa T, Kawaji H, et al Cap analysis gene expression for high-throughput analysis of transcriptional starting point and identification of promoter usage. Proc Natl Acad Sci U S A 2003;100:15776–81. 10.1073/pnas.2136655100 14663149 PMC307644

[15] McArthur E, Capra JA. Topologically associating domain boundaries that are stable across diverse cell types are evolutionarily constrained and enriched for heritability. Am J Hum Genet 2021;108:269–83. 10.1016/j.ajhg.2021.01.001 33545030 PMC7895846

[16] Herrmann W, Herrmann M. The importance of telomere shortening for atherosclerosis and mortality. J Cardiovasc Dev Dis 2020;7:29. 10.3390/jcdd7030029 32781553 PMC7570376

[17] Herman DS, Lam L, Taylor MRG, Wang L, Teekakirikul P, Christodoulou D, et al Truncations of titin causing dilated cardiomyopathy. N Engl J Med 2012;366:619–28. 10.1056/NEJMoa1110186 22335739 PMC3660031

[18] León P, Franco P, Hinojosa N, Torres K, Moreano A, Romero VI. TTN novel splice variant in familial dilated cardiomyopathy and splice variants review: a case report. Front Cardiovasc Med 2024;11:1387063. 10.3389/fcvm.2024.1387063 38938651 PMC11210389

[19] Samani NJ, Boultby R, Butler R, Thompson JR, Goodall AH. Telomere shortening in atherosclerosis. Lancet 2001;358:472–3. 10.1016/s0140-6736(01)05633-1 11513915

[20] Barra V, Fachinetti D. The dark side of centromeres: types, causes and consequences of structural abnormalities implicating centromeric DNA. Nat Commun 2018;9:4340. 10.1038/s41467-018-06545-y 30337534 PMC6194107

[21] Ting DT, Lipson D, Paul S, Brannigan BW, Akhavanfard S, Coffman EJ, et al Aberrant overexpression of satellite repeats in pancreatic and other epithelial cancers. Science 2011;331:593–6. 10.1126/science.1200801 21233348 PMC3701432

[22] Mendes de Almeida R, Tavares J, Martins S, Carvalho T, Enguita FJ, Brito D, et al Whole gene sequencing identifies deep-intronic variants with potential functional impact in patients with hypertrophic cardiomyopathy. PLoS One 2017;12:e0182946. 10.1371/journal.pone.0182946 28797094 PMC5552324

[23] Briganti F, Wang Z. Alternative splicing in the heart: the therapeutic potential of regulating the regulators. Int J Mol Sci 2024;25:13023. 10.3390/ijms252313023 39684734 PMC11641712

[24] Beqqali A . Alternative splicing in cardiomyopathy. Biophys Rev 2018;10:1061–71. 10.1007/s12551-018-0439-y 30051286 PMC6082314

[25] Freyermuth F, Rau F, Kokunai Y, Linke T, Sellier C, Nakamori M, et al Splicing misregulation of SCN5A contributes to cardiac-conduction delay and heart arrhythmia in myotonic dystrophy. Nat Commun 2016;7:11067. 10.1038/ncomms11067 27063795 PMC4831019

[26] Wood KA, Ellingford JM, Eden J, Thomas HB, O’Keefe RT, Hopton C, et al Pathogenic intronic splice-affecting variants in MYBPC3 in three patients with hypertrophic cardiomyopathy. Cardiogenetics 2021;11:73–83. 10.3390/cardiogenetics11020009

[27] Zhang L, Vincent GM, Baralle M, Baralle FE, Anson BD, Benson DW, et al An intronic mutation causes long QT syndrome. J Am Coll Cardiol 2004;44:1283–91. 10.1016/j.jacc.2004.06.045 15364333

[28] Rogozhina Y, Mironovich S, Shestak A, Adyan T, Polyakov A, Podolyak D, et al New intronic splicing mutation in the LMNA gene causing progressive cardiac conduction defects and variable myopathy. Gene 2016;595:202–6. 10.1016/j.gene.2016.10.001 27717888

[29] Dhandapany PS, Sadayappan S, Xue Y, Powell GT, Rani DS, Nallari P, et al A common MYBPC3 (cardiac myosin binding protein C) variant associated with cardiomyopathies in South Asia. Nat Genet 2009;41:187–91. 10.1038/ng.309 19151713 PMC2697598

[30] Du AY, Chobirko JD, Zhuo X, Feschotte C, Wang T. Regulatory transposable elements in the encyclopedia of DNA elements. Nat Commun 2024;15:7594. 10.1038/s41467-024-51921-6 39217141 PMC11366022

[31] Lawson HA, Liang Y, Wang T. Transposable elements in mammalian chromatin organization. Nat Rev Genet 2023;24:712–23. 10.1038/s41576-023-00609-6 37286742

[32] Mita P, Boeke JD. How retrotransposons shape genome regulation. Curr Opin Genet Dev 2016;37:90–100. 10.1016/j.gde.2016.01.001 26855260 PMC4914423

[33] Ma J, He D, Zhang M, Zhou Z, Cheng J, Huang A, et al Inhibition of long interspersed nuclear element-1 by nucleoside reverse transcriptase inhibitors attenuates vascular calcification. Signal Transduct Target Ther 2025;10:321. 10.1038/s41392-025-02396-4 41027876 PMC12484660

[34] Plaisance I, Chouvardas P, Sun Y, Nemir M, Aghagolzadeh P, Aminfar F, et al A transposable element into the human long noncoding RNA CARMEN is a switch for cardiac precursor cell specification. Cardiovasc Res 2023;119:1361–76. 10.1093/cvr/cvac191 36537036 PMC10262180

[35] Montasser ME, O'Hare EA, Wang X, Howard AD, McFarland R, Perry JA, et al An APOO pseudogene on chromosome 5q is associated with low-density lipoprotein cholesterol levels. Circulation 2018;138:1343–55. 10.1161/CIRCULATIONAHA.118.034016 29593015 PMC6162188

[36] Kim K, Theusch E, Kuang Y-L, Dose A, Mitchel K, Cubitt C, et al ZNF542P is a pseudogene associated with LDL response to simvastatin treatment. Sci Rep 2018;8:12443. 10.1038/s41598-018-30859-y 30127457 PMC6102286

[37] Lai Y, Li J, Zhong L, He X, Si X, Sun Y, et al The pseudogene PTENP1 regulates smooth muscle cells as a competing endogenous RNA. Clin Sci (Lond) 2019;133:1439–55. 10.1042/CS20190156 31235554

[38] Heckmann MB, Finke D, Sauerbrey L, Frey N, Lehmann LH. Increased expression of human endogenous retrovirus K in endomyocardial biopsies from patients with cardiomyopathy—a transcriptomics meta-analysis. BMC Genomics 2024;25:707. 10.1186/s12864-024-10595-6 39033293 PMC11264874

[39] Cheetham SW, Faulkner GJ, Dinger ME. Overcoming challenges and dogmas to understand the functions of pseudogenes. Nat Rev Genet 2020;21:191–201. 10.1038/s41576-019-0196-1 31848477

[40] Pink RC, Wicks K, Caley DP, Punch EK, Jacobs L, Carter DRF. Pseudogenes: pseudo-functional or key regulators in health and disease? RNA 2011;17:792–8. 10.1261/rna.2658311 21398401 PMC3078729

[41] Tian Y, Liu Y-F, Wang Y-Y, Li Y-Z, Ding W-Y, Zhang C. Molecular mechanisms of PTEN in atherosclerosis: a comprehensive review. Eur J Pharmacol 2024;979:176857. 10.1016/j.ejphar.2024.176857 39094923

[42] Lu S, Strand KA, Mutryn MF, Tucker RM, Jolly AJ, Furgeson SB, et al PTEN (Phosphatase and Tensin Homolog) protects against Ang II (Angiotensin II)-induced pathological vascular fibrosis and remodeling-brief report. Arterioscler Thromb Vasc Biol 2020;40:394–403. 10.1161/ATVBAHA.119.313757 31852223 PMC7059862

[43] Mattick JS, Amaral PP, Carninci P, Carpenter S, Chang HY, Chen L-L, et al Long non-coding RNAs: definitions, functions, challenges and recommendations. Nat Rev Mol Cell Biol 2023;24:430–47. 10.1038/s41580-022-00566-8 36596869 PMC10213152

[44] Mudge JM, Carbonell-Sala S, Diekhans M, Martinez JG, Hunt T, Jungreis I et al GENCODE 2025: reference gene annotation for human and mouse. Nucl Acid Res 2025;53:D966–75. 10.1093/nar/gkae1078 PMC11701607 39565199

[45] Vacante F, Rodor J, Lalwani MK, Mahmoud AD, Bennett M, De Pace AL, et al CARMN loss regulates smooth muscle cells and accelerates atherosclerosis in mice. Circ Res 2021;128:1258–75. 10.1161/CIRCRESAHA.120.318688 33622045 PMC7610708

[46] Michalik KM, You X, Manavski Y, Doddaballapur A, Zörnig M, Braun T, et al Long noncoding RNA MALAT1 regulates endothelial cell function and vessel growth. Circ Res 2014;114:1389–97. 10.1161/circresaha.114.303265 24602777

[47] Chen F, Li W, Zhang D, Fu Y, Yuan W, Luo G, et al MALAT1 regulates hypertrophy of cardiomyocytes by modulating the miR-181a/HMGB2 pathway. Eur J Histochem 2022;66:3426. 10.4081/ejh.2022.3426 35726535 PMC9251611

[48] Schober A, Nazari-Jahantigh M, Wei Y, Bidzhekov K, Gremse F, Grommes J, et al MicroRNA-126-5p promotes endothelial proliferation and limits atherosclerosis by suppressing Dlk1. Nat Med 2014;20:368–76. 10.1038/nm.3487 24584117 PMC4398028

[49] Zhang L, Zhou M, Qin G, Weintraub NL, Tang Y. MiR-92a regulates viability and angiogenesis of endothelial cells under oxidative stress. Biochem Biophys Res Commun 2014;446:952–8. 10.1016/j.bbrc.2014.03.035 24650666 PMC4016949

[50] Dong K, Shen J, He X, Hu G, Wang L, Osman I, et al CARMN is an evolutionarily conserved smooth muscle cell-specific LncRNA that maintains contractile phenotype by binding myocardin. Circulation 2021;144:1856–75. 10.1161/CIRCULATIONAHA.121.055949 34694145 PMC8726016

[51] Feng S, Qi Y, Xiao Z, Chen H, Liu S, Luo H, et al CircHIPK3 relieves vascular calcification via mediating SIRT1/PGC-1alpha/MFN2 pathway by interacting with FUS. BMC Cardiovasc Disord 2023;23:583. 10.1186/s12872-023-03602-3 38012555 PMC10683355

[52] Foinquinos A, Batkai S, Genschel C, Viereck J, Rump S, Gyöngyösi M, et al Preclinical development of a miR-132 inhibitor for heart failure treatment. Nat Commun 2020;11:633. 10.1038/s41467-020-14349-2 32005803 PMC6994493

[53] Gao X-Q, Zhang Y-H, Liu F, Ponnusamy M, Zhao X-M, Zhou L-Y, et al The piRNA CHAPIR regulates cardiac hypertrophy by controlling METTL3-dependent N(6)-methyladenosine methylation of Parp10 mRNA. Nat Cell Biol 2020;22:1319–31. 10.1038/s41556-020-0576-y 33020597

[54] Lim TB, Aliwarga E, Luu TDA, Li YP, Ng SL, Annadoray L, et al Targeting the highly abundant circular RNA circSlc8a1 in cardiomyocytes attenuates pressure overload induced hypertrophy. Cardiovasc Res 2019;115:1998–2007. 10.1093/cvr/cvz130 31114845

[55] Hullinger TG, Montgomery RL, Seto AG, Dickinson BA, Semus HM, Lynch JM, et al Inhibition of miR-15 protects against cardiac ischemic injury. Circ Res 2012;110:71–81. 10.1161/CIRCRESAHA.111.244442 22052914 PMC3354618

[56] Eken SM, Jin H, Chernogubova E, Li Y, Simon N, Sun C, et al MicroRNA-210 enhances fibrous cap stability in advanced atherosclerotic lesions. Circ Res 2017;120:633–44. 10.1161/CIRCRESAHA.116.309318 27895035

[57] Carè A, Catalucci D, Felicetti F, Bonci D, Addario A, Gallo P, et al MicroRNA-133 controls cardiac hypertrophy. Nat Med 2007;13:613–8. 10.1038/nm1582 17468766

[58] Viereck J, Kumarswamy R, Foinquinos A, Xiao K, Avramopoulos P, Kunz M, et al Long noncoding RNA Chast promotes cardiac remodeling. Sci Transl Med 2016;8:326ra22. 10.1126/scitranslmed.aaf1475 26888430

[59] Zou L, Ma X, Lin S, Wu B, Chen Y, Peng C. Long noncoding RNA-MEG3 contributes to myocardial ischemia-reperfusion injury through suppression of miR-7-5p expression. Biosci Rep 2019;39:BSR20190210. 10.1042/BSR20190210 31366567 PMC6702358

[60] Mahmoud AD, Ballantyne MD, Miscianinov V, Pinel K, Hung J, Scanlon JP, et al The human-specific and smooth muscle cell-enriched LncRNA SMILR promotes proliferation by regulating mitotic CENPF mRNA and drives cell-cycle progression which can be targeted to limit vascular remodeling. Circ Res 2019;125:535–51. 10.1161/CIRCRESAHA.119.314876 31339449 PMC6693924

[61] Brown SD, Malinowska AL, Bennett M, Correa-Sánchez AF, Horcasitas Valencia LL, Lucignoli AP, et al Small interfering RNA therapy targeting the long noncoding RNA SMILR for therapeutic intervention in coronary artery bypass graft failure. JACC Basic Transl Sci 2025;10:101364. 10.1016/j.jacbts.2025.101364 40928446 PMC12790151

[62] Ballantyne MD, Pinel K, Dakin R, Vesey AT, Diver L, Mackenzie R, et al Smooth Muscle Enriched Long Noncoding RNA (SMILR) regulates cell proliferation. Circulation 2016;133:2050–65. 10.1161/CIRCULATIONAHA.115.021019 27052414 PMC4872641

[63] Su Y, Zhu C, Wang B, Zheng H, McAlister V, Lacefield JC, et al Circular RNA Foxo3 in cardiac ischemia-reperfusion injury in heart transplantation: a new regulator and target. Am J Transplant 2021;21:2992–3004. 10.1111/ajt.16475 33382168

[64] Wang K, Zhou L-Y, Liu F, Lin L, Ju J, Tian P-C, et al PIWI-interacting RNA HAAPIR regulates cardiomyocyte death after myocardial infarction by promoting NAT10-mediated ac(4) C acetylation of Tfec mRNA. Adv Sci (Weinh) 2022;9:e2106058. 10.1002/advs.202106058 35138696 PMC8922123

[65] Yuan Q, Sun Y, Yang F, Yan D, Shen M, Jin Z, et al CircRNA DICAR as a novel endogenous regulator for diabetic cardiomyopathy and diabetic pyroptosis of cardiomyocytes. Signal Transduct Target Ther 2023;8:99. 10.1038/s41392-022-01306-2 36882410 PMC9992392

[66] Du WW, Yang W, Chen Y, Wu Z-K, Foster FS, Yang Z, et al Foxo3 circular RNA promotes cardiac senescence by modulating multiple factors associated with stress and senescence responses. Eur Heart J 2017;38:1402–12. 10.1093/eurheartj/ehw001 26873092

[67] Ma G, Bi S, Zhang P. Long non-coding RNA MIAT regulates ox-LDL-induced cell proliferation, migration and invasion by miR-641/STIM1 axis in human vascular smooth muscle cells. BMC Cardiovasc Disord 2021;21:248. 10.1186/s12872-021-02048-9 34016053 PMC8139145

[68] Cambier L, de Couto G, Ibrahim A, Echavez AK, Valle J, Liu W, et al Y RNA fragment in extracellular vesicles confers cardioprotection via modulation of IL-10 expression and secretion. EMBO Mol Med 2017;9:337–52. 10.15252/emmm.201606924 28167565 PMC5331234

[69] Ciullo A, Li L, Li C, Tsi K, Farrell C, Pellegrini M, et al Non-coding RNA yREX3 from human extracellular vesicles exerts macrophage-mediated cardioprotection via a novel gene-methylating mechanism. Eur Heart J 2024;45:2660–73. 10.1093/eurheartj/ehae357 38865332 PMC11297535

[70] Du F, Yu F, Wang Y, Hui Y, Carnevale K, Fu M, et al MicroRNA-155 deficiency results in decreased macrophage inflammation and attenuated atherogenesis in apolipoprotein E-deficient mice. Arterioscler Thromb Vasc Biol 2014;34:759–67. 10.1161/ATVBAHA.113.302701 24504735 PMC4036084

[71] Manea A, Vlad ML, Lazar AG, Manea SA. Focused ultrasound-induced targeted delivery of miR-155-5p inhibitor employing biomimetic microbubbles reduces NADPH oxidase expression and inflammation in atherosclerotic ApoE-/- mice. Cardiovasc Res 2022;118:cvac066-197. 10.1093/cvr/cvac066.197

[72] van Ingen E, van den Homberg DAL, van der Bent ML, Mei H, Papac-Milicevic N, Kremer V, et al C/D box snoRNA SNORD113-6/AF357425 plays a dual role in integrin signalling and arterial fibroblast function via pre-mRNA processing and 2'O-ribose methylation. Hum Mol Genet 2022;31:1051–66. 10.1093/hmg/ddab304 34673944 PMC8976432

[73] Micheletti R, Plaisance I, Abraham BJ, Sarre A, Ting C-C, Alexanian M, et al The long noncoding RNA Wisper controls cardiac fibrosis and remodeling. Sci Transl Med 2017;9:eaai9118. 10.1126/scitranslmed.aai9118 28637928 PMC5643582

[74] Fasolo F, Jin H, Winski G, Chernogubova E, Pauli J, Winter H, et al Long noncoding RNA MIAT controls advanced atherosclerotic lesion formation and plaque destabilization. Circulation 2021;144:1567–83. 10.1161/CIRCULATIONAHA.120.052023 34647815 PMC8570347

[75] Thum T, Gross C, Fiedler J, Fischer T, Kissler S, Bussen M, et al MicroRNA-21 contributes to myocardial disease by stimulating MAP kinase signalling in fibroblasts. Nature 2008;456:980–4. 10.1038/nature07511 19043405

[76] Sassi Y, Avramopoulos P, Ramanujam D, Grüter L, Werfel S, Giosele S, et al Cardiac myocyte miR-29 promotes pathological remodeling of the heart by activating Wnt signaling. Nat Commun 2017;8:1614. 10.1038/s41467-017-01737-4 29158499 PMC5696364

[77] Amaral P, Carbonell-Sala S, De La Vega FM, Faial T, Frankish A, Gingeras T, et al The status of the human gene catalogue. Nature 2023;622:41–7. 10.1038/s41586-023-06490-x 37794265 PMC10575709

[78] Shang R, Lee S, Senavirathne G, Lai EC. microRNAs in action: biogenesis, function and regulation. Nat Rev Genet 2023;24:816–33. 10.1038/s41576-023-00611-y 37380761 PMC11087887

[79] Caporali A, Anwar M, Devaux Y, Katare R, Martelli F, Srivastava PK, et al Non-coding RNAs as therapeutic targets and biomarkers in ischaemic heart disease. Nat Rev Cardiol 2024;21:556–73. 10.1038/s41569-024-01001-5 38499868

[80] Boon RA, Dimmeler S. MicroRNAs in myocardial infarction. Nat Rev Cardiol 2015;12:135–42. 10.1038/nrcardio.2014.207 25511085

[81] Bauersachs J, Solomon SD, Anker SD, Antorrena-Miranda I, Batkai S, Viereck J, et al Efficacy and safety of CDR132L in patients with reduced left ventricular ejection fraction after myocardial infarction: rationale and design of the HF-REVERT trial. Eur J Heart Fail 2024;26:674–82. 10.1002/ejhf.3139 38269451

[82] Täubel J, Hauke W, Rump S, Viereck J, Batkai S, Poetzsch J, et al Novel antisense therapy targeting microRNA-132 in patients with heart failure: results of a first-in-human phase 1b randomized, double-blind, placebo-controlled study. Eur Heart J 2021;42:178–88. 10.1093/eurheartj/ehaa898 33245749 PMC7954267

[83] Abplanalp WT, Fischer A, John D, Zeiher AM, Gosgnach W, Darville H, et al Efficiency and target derepression of anti-miR-92a: results of a first in human study. Nucleic Acid Ther 2020;30:335–45. 10.1089/nat.2020.0871 32707001

[84] Sallam T, van Solingen C, Moore KJ. Non-coding RNAs in lipid metabolism and their roles in atherosclerosis. Nat Rev Cardiol 2026;23:324–45. 10.1038/s41569-025-01229-9 41478885 PMC13525632

[85] Loyer X, Potteaux S, Vion A-C, Guérin CL, Boulkroun S, Rautou P-E, et al Inhibition of microRNA-92a prevents endothelial dysfunction and atherosclerosis in mice. Circ Res 2014;114:434–43. 10.1161/CIRCRESAHA.114.302213 24255059

[86] Eulalio A, Mano M, Dal Ferro M, Zentilin L, Sinagra G, Zacchigna S, et al Functional screening identifies miRNAs inducing cardiac regeneration. Nature 2012;492:376–81. 10.1038/nature11739 23222520

[87] Song R, Dasgupta C, Mulder C, Zhang L. MicroRNA-210 controls mitochondrial metabolism and protects heart function in myocardial infarction. Circulation 2022;145:1140–53. 10.1161/CIRCULATIONAHA.121.056929 35296158 PMC9007902

[88] Cullis PR, Felgner PL. The 60-year evolution of lipid nanoparticles for nucleic acid delivery. Nat Rev Drug Discov 2024;23:709–22. 10.1038/s41573-024-00977-6 38965378

[89] Wang S, Weissman D, Dong Y. RNA chemistry and therapeutics. Nat Rev Drug Discov 2025;24:828–51. 10.1038/s41573-025-01237-x 40659813 PMC12514552

[90] Håkansson KEJ, Goossens EAC, Trompet S, van Ingen E, de Vries MR, van der Kwast RVCT, et al Genetic associations and regulation of expression indicate an independent role for 14q32 snoRNAs in human cardiovascular disease. Cardiovasc Res 2019;115:1519–32. 10.1093/cvr/cvy309 30544252

[91] Valkov N, Das S. Y RNAs: biogenesis, function and implications for the cardiovascular system. Adv Exp Med Biol 2020;1229:327–42. 10.1007/978-981-15-1671-9_20 32285422 PMC7363058

[92] Zhang L, Liu J, Hou Y. Classification, function, and advances in tsRNA in non-neoplastic diseases. Cell Death Dis 2023;14:748. 10.1038/s41419-023-06250-9 37973899 PMC10654580

[93] Zeng Y, Jiang QL, Jiang J. Regulatory roles of mitochondrial tRNA-derived fragments in cardiovascular health and disease. J Am Heart Assoc 2025;14:e044656. 10.1161/JAHA.125.044656 41168945 PMC12684524

[94] Weick EM, Miska EA. piRNAs: from biogenesis to function. Development 2014;141:3458–71. 10.1242/dev.094037 25183868

[95] Hahne JC, Lampis A, Valeri N. Vault RNAs: hidden gems in RNA and protein regulation. Cell Mol Life Sci 2021;78:1487–99. 10.1007/s00018-020-03675-9 33063126 PMC7904556

[96] Bovia F, Strub K. The signal recognition particle and related small cytoplasmic ribonucleoprotein particles. J Cell Sci 1996;109 (Pt 11):2601–8. 10.1242/jcs.109.11.2601 8937977

[97] Peterlin BM, Brogie JE, Price DH. 7SK snRNA: a noncoding RNA that plays a major role in regulating eukaryotic transcription. Wiley Interdiscip Rev RNA 2012;3:92–103. 10.1002/wrna.106 21853533 PMC3223291

[98] Ramanathan A, Robb GB, Chan SH. mRNA capping: biological functions and applications. Nucleic Acids Res 2016;44:7511–26. 10.1093/nar/gkw551 27317694 PMC5027499

[99] Passmore LA, Coller J. Roles of mRNA poly(A) tails in regulation of eukaryotic gene expression. Nat Rev Mol Cell Biol 2022;23:93–106. 10.1038/s41580-021-00417-y 34594027 PMC7614307

[100] Statello L, Guo CJ, Chen LL, Huarte M. Gene regulation by long non-coding RNAs and its biological functions. Nat Rev Mol Cell Biol 2021;22:96–118. 10.1038/s41580-020-00315-9 33353982 PMC7754182

[101] Mably JD, Wang D-Z. Long non-coding RNAs in cardiac hypertrophy and heart failure: functions, mechanisms and clinical prospects. Nat Rev Cardiol 2024;21:326–45. 10.1038/s41569-023-00952-5 37985696 PMC11031336

[102] Zhang C, Niu K, Lian P, Hu Y, Shuai Z, Gao S, et al Pathological bases and clinical application of long noncoding RNAs in cardiovascular diseases. Hypertension 2021;78:16–29. 10.1161/hypertensionaha.120.16752 34058852

[103] Shah AM, Giacca M. Small non-coding RNA therapeutics for cardiovascular disease. Eur Heart J 2022;43:4548–61. 10.1093/eurheartj/ehac463 36106499 PMC9659475

[104] Holdt LM, Stahringer A, Sass K, Pichler G, Kulak NA, Wilfert W, et al Circular non-coding RNA ANRIL modulates ribosomal RNA maturation and atherosclerosis in humans. Nat Commun 2016;7:12429. 10.1038/ncomms12429 27539542 PMC4992165

[105] O'Leary E, Jiang Y, Kristensen LS, Hansen TB, Kjems J. The therapeutic potential of circular RNAs. Nat Rev Genet 2025;26:230–44. 10.1038/s41576-024-00806-x 39789148

[106] Si X, Zheng H, Wei G, Li M, Li W, Wang H, et al circRNA Hipk3 induces cardiac regeneration after myocardial infarction in mice by binding to Notch1 and miR-133a. Mol Ther Nucleic Acids 2020;21:636–55. 10.1016/j.omtn.2020.06.024 32736292 PMC7393325

[107] Thomson DW, Dinger ME. Endogenous microRNA sponges: evidence and controversy. Nat Rev Genet 2016;17:272–83. 10.1038/nrg.2016.20 27040487

[108] Huang C-K, Kafert-Kasting S, Thum T. Preclinical and clinical development of noncoding RNA therapeutics for cardiovascular disease. Circ Res 2020;126:663–78. 10.1161/CIRCRESAHA.119.315856 32105576 PMC7043728

[109] Fafard-Couture E, Bergeron D, Couture S, Abou-Elela S, Scott MS. Annotation of snoRNA abundance across human tissues reveals complex snoRNA-host gene relationships. Genome Biol 2021;22:172. 10.1186/s13059-021-02391-2 34088344 PMC8176728

[110] Ender C, Krek A, Friedländer MR, Beitzinger M, Weinmann L, Chen W, et al A human snoRNA with microRNA-like functions. Mol Cell 2008;32:519–28. 10.1016/j.molcel.2008.10.017 19026782

[111] Zhang Y, Deng Q, Tu L, Lv D, Liu D. tRNA-derived small RNAs: a novel class of small RNAs in human hypertrophic scar fibroblasts. Int J Mol Med 2020;45:115–30. 10.3892/ijmm.2019.4411 31939611 PMC6889923

[112] Di Fazio A, Schlackow M, Pong SK, Alagia A, Gullerova M. Dicer dependent tRNA derived small RNAs promote nascent RNA silencing. Nucleic Acids Res 2022;50:1734–52. 10.1093/nar/gkac022 35048990 PMC8860591

[113] Das S, Shah R, Dimmeler S, Freedman JE, Holley C, Lee J-M, et al Noncoding RNAs in cardiovascular disease: current knowledge, tools and technologies for investigation, and future directions: a scientific statement from the American Heart Association. Circulation 2020;13:e000062. 10.1161/HCG.0000000000000062 32812806

[114] Sahoo S, Adamiak M, Mathiyalagan P, Kenneweg F, Kafert-Kasting S, Thum T. Therapeutic and diagnostic translation of extracellular vesicles in cardiovascular diseases. Circulation 2021;143:1426–49. 10.1161/CIRCULATIONAHA.120.049254 33819075 PMC8021236

[115] Gilbert LA, Horlbeck MA, Adamson B, Villalta JE, Chen Y, Whitehead EH, et al Genome-scale CRISPR-mediated control of gene repression and activation. Cell 2014;159:647–61. 10.1016/j.cell.2014.09.029 25307932 PMC4253859

[116] Ghahremani S, Kanwal A, Pettinato A, Ladha F, Legere N, Thakar K, et al CRISPR activation reverses haploinsufficiency and functional deficits caused by TTN truncation variants. Circulation 2024;149:1285–97. 10.1161/CIRCULATIONAHA.123.063972 38235591 PMC11031707

[117] Mercer TR, Wilhelm D, Dinger ME, Soldà G, Korbie DJ, Glazov EA, et al Expression of distinct RNAs from 3´ untranslated regions. Nucleic Acids Res 2011;39:2393–403. 10.1093/nar/gkq1158 21075793 PMC3064787

[118] Malka Y, Steiman-Shimony A, Rosenthal E, Argaman L, Cohen-Daniel L, Arbib E, et al Post-transcriptional 3´-UTR cleavage of mRNA transcripts generates thousands of stable uncapped autonomous RNA fragments. Nat Commun 2017;8:2029. 10.1038/s41467-017-02099-7 29229900 PMC5725528

[119] Kocabas A, Duarte T, Kumar S, Hynes MA. Widespread differential expression of coding region and 3´ UTR sequences in neurons and other tissues. Neuron 2015;88:1149–56. 10.1016/j.neuron.2015.10.048 26687222

[120] Haberman N, Digby H, Faraway R, Cheung R, Chakrabarti AM, Jobbins AM, et al Widespread 3'UTR capped RNAs derive from G-rich regions in proximity to AGO2 binding sites. BMC Biol 2024;22:254. 10.1186/s12915-024-02032-7 39511645 PMC11546257

[121] Ng SB, Turner EH, Robertson PD, Flygare SD, Bigham AW, Lee C, et al Targeted capture and massively parallel sequencing of 12 human exomes. Nature 2009;461:272–6. 10.1038/nature08250 19684571 PMC2844771

[122] Ament IH, DeBruyne N, Wang F, Lin L. Long-read RNA sequencing: a transformative technology for exploring transcriptome complexity in human diseases. Mol Ther 2025;33:883–94. 10.1016/j.ymthe.2024.11.025 39563027 PMC11897757

[123] Shi J, Zhang Y, Li Y, Zhang L, Zhang X, Yan M, et al Optimized identification and characterization of small RNAs with PANDORA-seq. Nat Protoc 2025;20:3314–38. 10.1038/s41596-025-01158-4 40181099

[124] Core LJ, Waterfall JJ, Lis JT. Nascent RNA sequencing reveals widespread pausing and divergent initiation at human promoters. Science 2008;322:1845–8. 10.1126/science.1162228 19056941 PMC2833333

[125] Kwak H, Fuda NJ, Core LJ, Lis JT. Precise maps of RNA polymerase reveal how promoters direct initiation and pausing. Science 2013;339:950–3. 10.1126/science.1229386 23430654 PMC3974810

[126] Zhou J, Troyanskaya OG. Predicting effects of noncoding variants with deep learning-based sequence model. Nat Methods 2015;12:931–4. 10.1038/nmeth.3547 26301843 PMC4768299

[127] Avsec Ž, Agarwal V, Visentin D, Ledsam JR, Grabska-Barwinska A, Taylor KR, et al Effective gene expression prediction from sequence by integrating long-range interactions. Nat Methods 2021;18:1196–203. 10.1038/s41592-021-01252-x 34608324 PMC8490152

[128] Mangul S, Martin LS, Hill BL, Lam AK-M, Distler MG, Zelikovsky A, et al Systematic benchmarking of omics computational tools. Nat Commun 2019;10:1393. 10.1038/s41467-019-09406-4 30918265 PMC6437167

[129] Litviňuková M, Talavera-López C, Maatz H, Reichart D, Worth CL, Lindberg EL, et al Cells of the adult human heart. Nature 2020;588:466–72. 10.1038/s41586-020-2797-4 32971526 PMC7681775

[130] Isakova A, Liu DD, Cvijović I, Sinha R, Eastman AE, Saul S, et al Scalable single-cell total RNA sequencing unifies coding and noncoding transcriptomics. Nat Biotechnol 2026;44. 10.1038/s41587-026-03068-6. PMC13268204 41917462

[131] Walsh R, Jurgens SJ, Erdmann J, Bezzina CR. Genome-wide association studies of cardiovascular disease. Physiol Rev 2023;103:2039–55. 10.1152/physrev.00024.2022 36634218

[132] Johnsson P, Lipovich L, Grander D, Morris KV. Evolutionary conservation of long non-coding RNAs; sequence, structure, function. Biochim Biophys Acta 2014;1840:1063–71. 10.1016/j.bbagen.2013.10.035 24184936 PMC3909678

[133] Edwards SL, Beesley J, French JD, Dunning AM. Beyond GWASs: illuminating the dark road from association to function. Am J Hum Genet 2013;93:779–97. 10.1016/j.ajhg.2013.10.012 24210251 PMC3824120

[134] Baker AH, Giacca M, Thum T. miRNA discovery to therapy: the field is sufficiently mature to assess the value of miRNA-based therapeutics. Mol Ther 2025;33:3–4. 10.1016/j.ymthe.2024.12.024 39708798 PMC11764860

[135] Khera AV, Chaffin M, Aragam KG, Haas ME, Roselli C, Choi SH, et al Genome-wide polygenic scores for common diseases identify individuals with risk equivalent to monogenic mutations. Nat Genet 2018;50:1219–24. 10.1038/s41588-018-0183-z 30104762 PMC6128408

[136] Saghatelian A, Couso JP. Discovery and characterization of smORF-encoded bioactive polypeptides. Nat Chem Biol 2015;11:909–16. 10.1038/nchembio.1964 26575237 PMC4956473

[137] Nelson BR, Makarewich CA, Anderson DM, Winders BR, Troupes CD, Wu F, et al A peptide encoded by a transcript annotated as long noncoding RNA enhances SERCA activity in muscle. Science 2016;351:271–5. 10.1126/science.aad4076 26816378 PMC4892890

[138] Makarewich CA, Baskin KK, Munir AZ, Bezprozvannaya S, Sharma G, Khemtong C, et al MOXI is a mitochondrial micropeptide that enhances fatty acid β-oxidation. Cell Rep 2018;23:3701–9. 10.1016/j.celrep.2018.05.058 29949755 PMC6066340

[139] Spencer HL, Sanders R, Boulberdaa M, Meloni M, Cochrane A, Spiroski A-M, et al The LINC00961 transcript and its encoded micropeptide, small regulatory polypeptide of amino acid response, regulate endothelial cell function. Cardiovasc Res 2020;116:1981–94. 10.1093/cvr/cvaa008 31990292 PMC8216332

[140] Yan Y, Zhang T, He X, Du T, Dai G, Xu X, et al A cardiac fibroblast-enriched micropeptide regulates inflammation in ischemia/reperfusion injury. JCI Insight 2025;10:e187848. 10.1172/jci.insight.187848 40111415 PMC12128956

[141] Liu X, Chen S, Luo W, Yu C, Yan S, Lei L, et al LncRNA MFRL regulates the phenotypic switch of vascular smooth muscle cells to attenuate arterial remodeling by encoding a novel micropeptide MFRLP. Transl Res 2024;272:54–67. 10.1016/j.trsl.2024.05.009 38838852

[142] Engreitz JM, Haines JE, Perez EM, Munson G, Chen J, Kane M, et al Local regulation of gene expression by lncRNA promoters, transcription and splicing. Nature 2016;539:452–5. 10.1038/nature20149 27783602 PMC6853796

[143] Holdt LM, Hoffmann S, Sass K, Langenberger D, Scholz M, Krohn K, et al Alu elements in ANRIL non-coding RNA at chromosome 9p21 modulate atherogenic cell functions through trans-regulation of gene networks. PLoS Genet 2013;9:e1003588. 10.1371/journal.pgen.1003588 23861667 PMC3701717

[144] Nurk S, Koren S, Rhie A, Rautiainen M, Bzikadze AV, Mikheenko A, et al The complete sequence of a human genome. Science 2022;376:44–53. 10.1126/science.abj6987 35357919 PMC9186530

[145] Paldi F, Cavalli G. 3D genome folding in epigenetic regulation and cellular memory. Trends Cell Biol 2026;36:28–41. 10.1016/j.tcb.2025.03.001 40221344 PMC7619243

