# Supplementary material for: The dark genome in cardiovascular medicine
Source: Eur Heart J. 2026 Jul 9;47(33):4574–92. doi: 10.1093/eurheartj/ehag514 (PMC13533564; doi:10.1093/eurheartj/ehag514)
Supplement: Supplementary file 1 [file ehag514_supplementary_data.zip › Supplementary Table 1.docx]

**Table 1. Glossary of key terms relevant to the dark genome and non-coding RNA biology.** Terms are listed in alphabetical order, together with concise definitions and their relevance to the context of this review.

| **Term** | **Definition** | **Context in this review** |
| --- | --- | --- |
| **Alu element / Alu-derived RNA** | A common primate transposable element; can be transcribed into RNAs/fragments. | Illustrates repeat-associated transcription and immune/stress links. |
| **Back-splicing** | Splicing event that joins a downstream exon to an upstream exon to form a circle. | Mechanistic basis for circRNA biogenesis. |
| **Chromatin** | DNA packaged with proteins; its “state” affects gene activity. | Many non-coding RNAs act by influencing chromatin and transcription. |
| **Circular RNA (circRNA)** | RNA made by back-splicing into a covalently closed circle. | Often more stable; potential biomarkers and therapeutic targets in disease. |
| **Cis-acting** | Acting near the site where the RNA is transcribed (local effects). | Key concept for lncRNA mechanism and experimental design. |
| **CRISPR activation (CRISPRa)** | CRISPR-based activation of transcription. | Useful to test gain-of-function at non-coding RNA loci. |
| **CRISPR interference (CRISPRi)** | CRISPR-based repression of transcription without cutting DNA. | Often better for cis-acting lncRNA loci than RNA knockdown alone. |
| **Dark genome** | All genome sequence in an organism that does not encode canonical protein or their regulatory DNA and associated RNA transcripts. | The conceptual umbrella for the review. |
| **Dark proteome** | Proteins/peptides not well captured by traditional annotations or by proteomic methodologies. | Frames micropeptides as an emerging layer of important biology. |
| **Enhancer** | DNA element that increases transcription, often from a genetic distance away from the gene. | Important for cell-type specificity in transcription; often implicated by genome-wide association studies (GWAS). |
| **Exon** | A segment retained in mature RNA after splicing; In coding genes exons include the protein-coding sequence and 5’ and 3’ untranslated regions. | Protein-coding exons are a small fraction of the genome (~1–2%). |
| **Extracellular vesicles (EVs)** | Membrane-bound particles carrying RNAs/proteins between cells. | Important route for ncRNA biomarkers and signalling in CVD. |
| **Functional perturbation** | Experimental disruption (e.g., knockdown, CRISPRi/a) to test function. | Needed to move from association to causation. |
| **Gene locus** | A defined genomic location | Many gene loci produce regulatory RNA transcripts and, in some cases, also produce protein-coding isoforms or micropeptides |
| **Genome** | The complete DNA sequence of an organism. | Sets the context for “protein coding” versus “non-coding” function. |
| **Genome-wide association studies (GWAS)** | Studies linking common genetic variants to traits/diseases. | Many disease-associated signals map to non-coding regions. |
| **Insulator** | DNA element that constrains enhancer–promoter communication (often via chromatin architecture). | Relevant to long-range gene regulation. |
| **Intergenic region** | DNA sequences between annotated genes. | Rich source of regulatory elements and non-coding RNAs. |
| **Intron** | A segment removed from RNA during RNA splicing. | Many non-coding RNAs arise from introns within other genes (including protein-coding and other non-coding RNAs like lncRNAs) |
| **Isoform** | Different transcript versions from the same gene locus (often created via alternative splicing). | Relevant for interpreting transcript diversity and functional annotation of lncRNAs |
| **Long non-coding RNA** | Non-coding RNAs >500 nucleotides in length | RNAs with diverse mechanisms: cis/trans, nuclear/cytoplasmic, scaffolds/decoys/guides. |
| **Long-read sequencing** | Sequencing that reads full-length RNA molecules more directly. | Improves isoform annotation for long non-coding RNAs/circRNAs. |
| **Micropeptides** | Short proteins, typically <100–150 amino acids, translated from small open reading frames (smORFs), often within transcripts previously annotated as non-coding RNAs or untranslated regions. | Illustrate that parts of the dark genome can give rise to biologically active peptides, blurring the distinction between coding and non-coding loci and expanding the functional output of the dark genome. |
| **MicroRNA (miRNA)** | ~22 nucleotide RNAs that guide silencing complexes to target RNAs. | Strong evidence base for function in cardiovascular health and disease. |
| **Nascent transcription** | Newly synthesised RNA (often unstable). | Helps detect promoter/enhancer-associated transcription. |
| **Non-coding RNA (ncRNA)** | RNA not translated into canonical proteins (though some loci may still encode micropeptides). | A major functional output of the dark genome. |
| **Non-coding variant** | A DNA variant outside protein-coding exons. | Major interpretive challenge; key translational opportunity. |
| **Poly(A) tail / polyadenylation** | A tail of adenine nucleotides added to many RNAs after transcription of DNA to RNA; exploited by common RNA-seq methods. | Determines what standard RNA-seq captures (and misses). |
| **Poly(A)-selected RNA-seq** | RNA-seq that enriches polyadenylated RNAs. | Can capture many lncRNAs; misses many non-poly(A) non-coding RNA species. |
| **Promoter** | DNA region where transcription starts for a gene. | Many disease variants sit in regulatory DNA in and around gene promoters. |
| **Pseudogene** | Gene-like sequence usually not producing a functional protein. | Can exert regulatory effects. |
| **Regulatory network** | Interacting set of genes/RNAs/proteins controlling cell state. | Helps frame non-coding RNAs as systems-level regulators. |
| **Ribosomal RNA (rRNA)** | Structural/catalytic RNAs forming the ribosome. | Long non-coding RNAs included for completeness, not the main “regulatory” focus. |
| **RNA modifications** | Chemical marks on RNA (e.g., methylation/acetylation) that affect function/measurement. | Especially relevant for tRNAs/tsRNAs and some small RNAs. |
| **Silencer** | DNA element that reduces transcription. | Provides a mechanism for reduced gene transcription independent of changes to protein-coding sequences |
| **Small non-coding RNA** | Non-coding RNAs <500 nucleotides in length | Includes miRNAs, snoRNAs, tsRNAs, Y RNAs etc. |
| **Small nuclear RNA (snRNA)** | RNAs that form the spliceosome. | Links ncRNAs to splicing control in disease. |
| **Small nucleolar RNA (snoRNA)** | Small non-coding RNAs that guide site-specific chemical modification (e.g. methylation or pseudouridylation) of other RNAs, primarily ribosomal RNAs and small nuclear RNAs. | Emerging cardiovascular roles; also produce fragments in some contexts. |
| **Spliceosome** | A ribonucleoprotein complex composed of small nuclear RNAs (snRNAs) and associated proteins that catalyses the removal of introns from precursor messenger RNAs (pre-mRNAs) during splicing. | Links small nuclear RNAs to transcript processing and highlights RNA-based regulation of gene expression at the splicing level |
| **Total RNA-seq (rRNA-depleted)** | RNA-seq after removing rRNA to profile a broad RNA pool. | Better for circRNAs and many non-poly(A) RNAs. |
| **Trans-acting** | Acting away from its transcription site (distant loci or cytoplasm). | Explains broader, network-level lncRNA effects. |
| **Transcription** | The process of generating RNA from DNA. | “Non-coding” does not mean “not transcribed”. |
| **Transcription start site (TSS)** | The nucleotide location where transcription of RNAs begins. | Important for annotating gene promoters and regulatory regions that impact gene transcription. |
| **Translation** | The process of generating protein from RNA. | Distinguishes non-coding RNA from protein. |
| **Transposable element (TE)** | Repetitive DNA that can copy/insert in the genome. | A major component of the dark genome. |
| **tRNA (transfer RNA)** | RNAs that deliver specific amino acids to the ribosome during protein synthesis (“translation”). | Source molecules for tsRNAs; link to stress responses. |
| **tRNA-derived small RNA (tsRNA)** | Small RNAs processed from tRNAs, often stress-associated. | Implicated in regulation of translation and cellular stress responses |
| **Untranslated region (UTR)** | Parts of an mRNA not translated into protein (5′ UTR, 3′ UTR). | Major sites of post-transcriptional control. |
| **Y RNA** | Structured small RNAs with protein partners; can generate bioactive fragments. | Increasing cardiovascular and extracellular vesicle (EV) literature to support functional importance. |
